# NLRP3 Inflammasome and IL-33: Novel Players in Sterile Liver Inflammation

**DOI:** 10.3390/ijms19092732

**Published:** 2018-09-12

**Authors:** Katrin Neumann, Birgit Schiller, Gisa Tiegs

**Affiliations:** Institute of Experimental Immunology and Hepatology, University Medical Center Hamburg-Eppendorf, 20246 Hamburg, Germany; b.schiller@uke.de (B.S.); g.tiegs@uke.de (G.T.)

**Keywords:** sterile liver inflammation, NLRP3 inflammasome, IL-1β, IL-33, ILC2, tregs

## Abstract

In sterile liver inflammation, danger signals are released in response to tissue injury to alert the immune system; e.g., by activation of the NLRP3 inflammasome. Recently, IL-33 has been identified as a novel type of danger signal or “alarmin”, which is released from damaged and necrotic cells. IL-33 is a pleiotropic cytokine that targets a broad range of immune cells and exhibits pro- and anti-inflammatory properties dependent on the disease. This review summarizes the immunomodulatory roles of the NLRP3 inflammasome and IL-33 in sterile liver inflammation and highlights potential therapeutic strategies targeting these pathways in liver disease.

## 1. Introduction

Sterile inflammation occurs in response to tissue injury and cell death in the absence of pathogens and triggers processes of regeneration and wound repair to maintain tissue homeostasis. A variety of stimuli induce sterile inflammation including toxins, antigens, mechanical trauma, and ischemia [1]. Importantly, if the sterile stimulus is not resolved, this drives chronic inflammation, extensive tissue damage as well as fibrogenesis and can induce or aggravate a variety of diseases. Sterile inflammation has been identified as a main component of the pathologies of nonalcoholic steatohepatitis (NASH), alcoholic steatohepatitis, drug-induced liver injury, liver ischemia reperfusion (I/R), and autoimmune hepatitis (AIH). In these sterile inflammation-associated liver diseases, different danger signals, called damage-associated molecular patterns (DAMPs), are released from damaged and dying cells and induce sterile liver inflammation; e.g., by activation of inflammasomes [2]. Sterile inflammation-associated liver diseases are a severe health problem in the industrialized world with very limited therapeutic options so far. Thus, understanding cellular and molecular mechanisms that drive sterile inflammation will provide the opportunity to selectively target pathways involved in sterile liver diseases. In this review, we will summarize the current knowledge about the inflammatory function of the NLRP3 inflammasome in sterile inflammation-associated liver disease. We will further provide an overview on the immunoregulatory role of a special type of DAMP or alarmin in sterile liver inflammation-the cytokine interleukin (IL)-33.

## 2. Sterile Inflammation-Associated Liver Diseases

### 2.1. Nonalcoholic Fatty Liver Disease

Nonalcoholic fatty liver disease (NAFLD) is a result of overnutrition characterized by lipid accumulation in hepatocytes in the absence of excessive alcohol consumption [3]. NAFLD is often associated with obesity, hyperlipidemia, and insulin resistance [4], and can manifest as hepatic steatosis without inflammation or fibrosis or as NASH, which is accompanied by cellular damage, inflammation, and fibrosis that can progress to cirrhosis and hepatocellular carcinoma [5,6]. A two-hit hypothesis has been proposed to describe the mechanisms driving the progression of NASH. The first hit is induced by lipid accumulation and lipotoxicity causing hepatocyte injury, but a second hit is required for constant liver damage and includes inflammation, oxidative stress, mitochondrial dysfunction, and lipid peroxidation [7]. It has been further suggested that underlying steatosis sensitizes the liver to stressors of the second hit thereby facilitating progression of NASH in the steatotic liver [8,9].

### 2.2. Alcoholic Liver Disease

Excessive alcohol consumption leads to chronic liver disease. The clinical spectrum of alcoholic liver disease (ALD) includes steatosis, steatohepatitis, cirrhosis, and hepatocellular carcinoma [10]. The current hypothesis for alcohol-induced liver injury suggests that constant ethanol exposure results in leakage of bacterial products from the gut and alters the gut microflora leading to an increase in Gram-negative bacteria and serum lipopolysaccharide (LPS) levels in alcoholics [11]. Moreover, alcohol-induced oxidative stress [12] and activation of the immune system [13,14] have been recognized to cause liver injury. Alcohol regulates transcription factors related to lipid metabolism, promotes fatty acid synthesis, and inhibits fatty acid oxidation leading to lipid accumulation in the liver. Hepatocyte dysfunction, infiltration of inflammatory monocytes, and activation of liver resident macrophages (Kupffer cells) are central to the pathogenesis of alcoholic steatohepatitis [15].

### 2.3. Drug-Induced Liver Injury

Drug-induced liver injury has been linked to over 1000 drugs and is a frequent cause of acute liver failure. Clinical manifestations of drug-induced liver injury are acute hepatitis, cholestasis, jaundice, or sinusoidal obstruction syndrome [16,17]. Disease severity depends on the drug, its concentration and frequency of intake, its metabolization in the liver, but also on the patient’s age, sex, and body-mass index [18,19,20]. Drug-induced liver injury is induced by direct hepatotoxic effects of a drug or a reactive metabolite of a drug, which mediates protein dysfunction, lipid peroxidation, DNA damage, and oxidative stress. This triggers hepatocyte death and the initiation of innate immune responses. The analgesic acetaminophen (APAP) is a drug with hepatotoxic effects that rapidly causes hepatocyte injury and development of necrosis. APAP overdose has been identified as one of the main reasons for acute liver failure [16,17].

### 2.4. Liver Ischemia/Reperfusion (I/R) Injury

Liver I/R is a process that involves deprivation of blood flow and oxygen followed by their restoration, leading to ischemic organ damage and inflammation-mediated reperfusion injury [21]. Hepatic I/R injury is a clinical complication of partial hepatectomy, liver transplantation, and trauma. The hepatic immune response in I/R injury comprises two phases. In the first phase, the ischemic insult causes oxidative stress and the production of reactive oxygen species (ROS) within the liver resulting in Kupffer cell activation and oxidant-mediated hepatocyte death [22,23,24]. In the second phase, reperfusion promotes injury by driving sterile inflammation, release of chemokines, inflammatory cytokines, and infiltration of neutrophils [25,26]. Inflammation in liver I/R is initiated by activated Kupffer cells that phagocytose necrotic hepatocytes, secrete inflammatory cytokines, and recruit other immune cell populations, such as neutrophils and inflammatory monocytes [21,27].

### 2.5. Autoimmune Hepatitis (AIH)

AIH is an autoimmune liver disease present in acute and chronic forms. In AIH, loss of tolerance against liver autoantigens leads to elevated serum aminotransferase levels, presence of non-organ-specific autoantibodies with unknown function, hyperglobulinemia, progressive destruction of the hepatic parenchyma, and development of liver fibrosis. Chronic AIH can progress to liver cirrhosis, fulminant hepatic failure, and liver cancer [28,29,30]. The exact mechanisms leading to immunity against hepatic autoantigens have not been fully clarified. However, impaired immune regulation by regulatory T cells (Treg) and differentiation of inflammatory Th17 cells have been suggested to play a central role [28,31,32].

## 3. The Inflammasome

### 3.1. Inflammasome Function

Sterile inflammation amplifies liver damage by activation of inflammasomes, which are cytosolic multiprotein complexes essential for a rapid response of the innate immune system to the presence of pathogen-associated molecular pattern (PAMPs), derived from invading pathogens, and damage-associated molecular pattern (DAMPs) induced as a result of endogenous stress [33]. Recognition of PAMPs and DAMPs by germline-encoded pattern-recognition receptors expressed by innate immune cells leads to inflammasome activation resulting in activation of the protease caspase-1 and secretion of the pro-inflammatory cytokines interleukin (IL)-1β and IL-18 [34] that play a critical role in liver disease. Inflammasome activation can also cause pyroptosis, an inflammatory cell death associated with cellular lysis and release of intracellular content into the extracellular space [35]. In the absence of pathogens, DAMP-induced inflammation is termed sterile inflammation [33].

### 3.2. Inflammasome Activation

Full inflammasome activation requires two distinct signals. First, recognition of Toll-like receptor (TLR) ligands, such as lipopolysaccharide (LPS), through their respective TLRs or binding of the cytokines tumor necrosis factor α (TNFα) and IL-1β to their cytokine receptors induces activation of the NF-κB pathway and production of pro-IL-1β and pro-IL-18 precursors. In the absence of a second signal, the cytokine precursors are inactive and remain inside the cell. The second signal is induced by a variety of endogenous DAMPs including adenosine triphosphate (ATP), reactive oxygen species (ROS), high mobility group box 1 (HMGB1) protein, host DNA, uric acid, and cholesterol crystals [34,36,37]. These DAMPs are detected by a family of pattern-recognition molecules termed nucleotide-binding oligomerization domain-like receptor (NLR) [38]. Each of the NLR members can assemble a complex with a caspase-recruiting domain thereby generating a protein complex responsible for binding and autoproteolytic cleavage of pro-caspase-1 into active caspase-1 that in turn cleaves pro-IL-1β and pro-IL-18 to mature IL-1β and IL-18, which enables secretion of these cytokines [39,40,41]. Of note, DAMPs are also recognized by receptors that are usually activated by PAMPs, for example TLRs [42]. Others, like the purinergic P2X7 receptor, appear to be uniquely activated by DAMPs [43].

### 3.3. IL-1β and NLRP3 Inflammasome

In principle, there are different inflammasome complexes named by the protein forming the scaffold (e.g., NLRP3, NLRC4, AIM2) but ultimately, all regulate activation of caspase-1 and secretion of mature IL-1β [34,44]. IL-1β is a potent pro-inflammatory cytokine that plays a key role in innate and adaptive immunity. IL-1β recruits IL-1 receptor (IL-1R)-expressing effector cells like neutrophils and monocytes and induces Th17-cell differentiation, thereby amplifying inflammation and autoimmune diseases [45,46]. One well studied inflammasome is the NLRP3 inflammasome, which contributes to the development of sterile liver inflammation in ALD and NAFLD [47,48,49]. Hepatic NLRP3 activation is achieved by different DAMPs, among others by release of intracellular ATP in response to cell death, hypoxia/ischemia, and pathogen infection [50]. Extracellular ATP levels are tightly regulated by ectonucleotidases, particularly CD39, which converts ATP to adenosine monophosphate [51,52]. Extracellular ATP stimulates the purinergic P2X7 receptor that induces NLRP3 inflammasome activation through a decrease in intracellular potassium levels or through generation of ROS [53,54,55].

Overall, inflammasome activation induces a strong pro-inflammatory response and therefore must be well regulated. There is an increasing body of evidence that inflammasome dysregulation drives pathogenesis in diseases like multiple sclerosis, obesity, type 2 diabetes, Alzheimer’s disease, atherosclerosis, inflammatory bowel disease, and tumorigenesis [56,57,58]. Consequences of NLRP3 hyperactivation were illustrated by using *Nlrp3* knock-in mice that were seriously impaired in growth and survival and developed severe liver inflammation and fibrosis associated with hepatocyte pyroptosis, neutrophil infiltration, and hepatic stellate cell (HSC)-mediated collagen deposition [59]. Thus, understanding inflammasome activation and regulation will provide the possibility to therapeutically interfere with processes of inflammation and fibrosis in the liver.

## 4. NLRP3 Inflammasome Activation in Sterile Inflammation-Associated Liver Diseases

The concept of DAMPs driving inflammation has been intensively investigated in sterile liver disease. During tissue damage, dying hepatocytes release DAMPs that act on neighboring cells to trigger inflammation and tissue regeneration. Persistence of these signals in chronic disease aggravates inflammation and leads to excessive scarring and loss of liver function. Inflammasome components were identified in hepatocytes, Kupffer cells, liver sinusoidal endothelial cells (LSEC) and HSC [60,61]. In this section, we will focus on the NLRP3 inflammasome and summarize the recent findings of its function in sterile liver disease. An overview of inflammasome components in sterile liver disease and their potential as therapeutic targets are listed in Table 1 and Table 2, respectively.

### 4.1. NAFLD

Several studies have revealed that the NLRP3 inflammasome drives sterile inflammation in NAFLD. In NASH patients, increased NLRP3 [62,63] and IL-1β levels were shown and positively correlated with liver fibrosis [64]. Elevated NLRP3 and mature IL-1β levels were also demonstrated in different murine diet-induced steatohepatitis models [63,65,66]. Moreover, *Nlrp3*^−/−^ mice showed marked protection from liver injury and fibrosis whereas tamoxifen-induced *Nlrp3* knock-in mice were characterized by accelerated hepatic injury and fibrogenesis [62,64,67]. Further, pharmacological inhibition of the NLRP3 inflammasome by the inhibitor BAY 11-7082 reduced caspase-1 activation as well as IL-1β production, and ameliorated diet-induced steatohepatitis [99]. Progression of NASH was associated with NLRP3 inflammasome activation in hepatocytes [63], and activation of caspase-1 in Kupffer cells [68]. Strikingly, both *Casp1*^−/−^ mice and Kupffer cell-depleted wildtype mice showed less severe diet-induced steatohepatitis [68,69] indicating that caspase-1 in Kupffer cells plays an important role in the pathogenesis of NASH.

Activation of the inflammasome pathway in NASH can be mediated by extracellular ATP since P2X7 receptor-deficient (*P2rx7*^−/−^) mice developed markedly reduced liver fibrosis in diet-induced steatohepatitis [70]. Moreover, free cholesterol, a lipotoxic lipid present in NASH patients and mice with diet-induced steatohepatitis, was shown to promote NASH progression [100,101,102]. High levels of free cholesterol lead to cholesterol crystal formation, which act as DAMP and activate the NLRP3 inflammasome [103,104]. Cholesterol crystals were found in steatotic hepatocytes of patients with NASH but not steatosis and in mice with diet-induced steatohepatitis [48,71]. One study reported activation of the NLRP3 inflammasome and caspase-1 in Kupffer cells surrounding cholesterol crystal-containing hepatocytes thereby linking lipotoxicity with inflammation. Moreover, exposure of HepG2 cells to palmitic acid was shown to induce lipotoxicity and release of microvesicles, which in turn triggered NLRP3 inflammasome activation and IL-1β release after internalization by macrophages [105]. As an interesting therapeutic approach, the cholesterol-lowering drug ezetimibe was used leading to reduced cholesterol crystal formation and fibrosis [96]. Blockage of NLRP3 inflammasome activation in Kupffer cells through the small molecule inhibitor MCC950 was tested as another therapeutic option in NASH. MCC950 did not prevent cholesterol crystal formation and development of steatosis but inhibited Kupffer cell activation and reduced inflammation and fibrosis [48,72].

In NASH pathology, not only DAMPs but also PAMPs play a role and it has been proposed that both act together and worsen liver injury. Free fatty acids, which are elevated in NASH patients [106], have been suggested to function as DAMPs and together with TLR ligands activate the NLRP3 inflammasome [63,107]. Palmitic acid in conjunction with the TLR4 ligand LPS or the synthetic TLR2 ligand Pam3CK4 was shown to cooperatively induce NLRP3 inflammasome activation in hepatocytes and Kupffer cells, respectively, whereas the fatty acid alone did not activate the NLRP3 inflammasome [63,107]. In contrast, a direct palmitic acid-induced NLRP3 inflammasome activation and subsequent IL-1β production in Kupffer cells was also reported [67]. It has been further suggested that free fatty acids indirectly trigger NLRP3 inflammasome activation by inducing cell death [108]. One study correlated palmitic acid-induced hepatocyte death with up-regulation of NLRP3 inflammasome and IL-1β levels and indicated that DAMPs, released from dying hepatocytes, rather than free fatty acids drive NLRP3 inflammasome activation in NASH [63]. 

### 4.2. ALD

NLRP3/caspase-1 mediated induction of IL-1β expression in Kupffer cells was identified as a driver of ALD pathology in murine model of chronic ethanol consumption and pharmacological blockade of IL-1R signaling was proven to be beneficial for alcohol-induced liver injury [47]. A pathogenic role of this cytokine was also suggested for ALD patients that showed elevated IL-1β levels [73,74]. Kupffer cell-derived IL-1β was found to trigger activation and recruitment of invariant NKT cells, which in turn drive liver inflammation by expressing the pro-inflammatory cytokine TNFα and recruiting inflammatory neutrophils. In this study, iNKT cell-deficient *Jα18*^−/−^ mice and *Nlrp3*^−/−^ mice were used and both developed less severe ethanol-induced liver damage and steatosis [75]. The fact that NLRP3 inflammasome activation is crucial for ALD progression was reproduced by a follow up study [76], however, another study proposed a protective role since in this case, *Nlrp3*^−/−^ mice were more susceptible to ethanol-induced liver injury [109].

**Figure 1:**
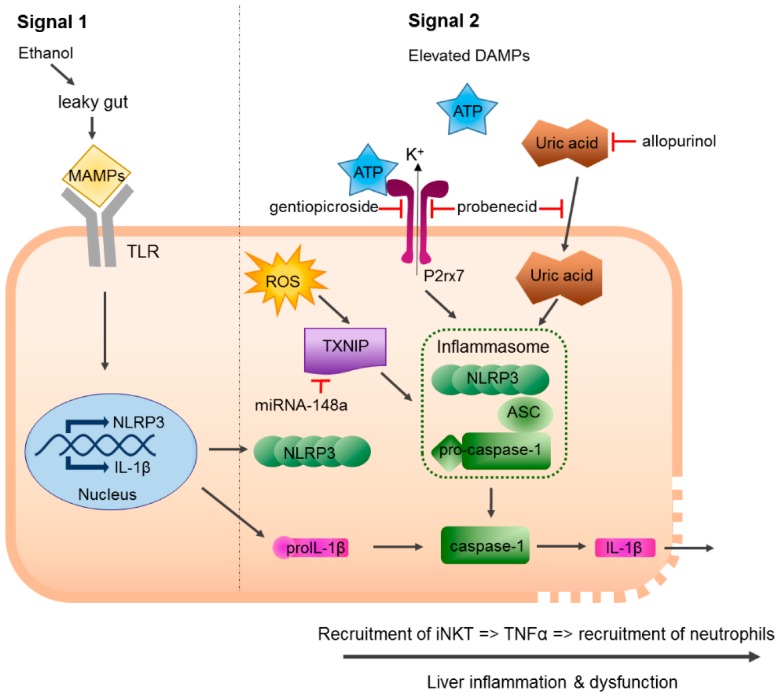
Mechanisms of NLRP3 inflammasome activation and inhibition in alcoholic liver disease (ALD). Constant ethanol exposure results in leakage of bacterial products, termed as microbe-associated molecular pattern, from the gut that trigger TLR-mediated signal 1. Extracellular ATP, uric acid, and ROS are elevated DAMPs in ALD and induce signal 2 leading to NLRP3 inflammasome activation and cleavage of pro-caspase-1 into active caspase-1, which in turn cleaves pro-IL-1β into mature IL-1β thereby enabling secretion of this inflammatory cytokine. Released IL-1β induces activation and recruitment of iNKT cells that produce TNFα and recruit inflammatory neutrophils. NLRP3 inflammasome activation and IL-1β release can be prevented by substances that inhibit P2rx7-mediated signaling as well as miRNA-148a, which is a suppressor of TXNIP required for inflammasome activation. MAMPs: microbe-associated molecular pattern, DAMPs, danger-associated molecular pattern, TXNIP, thioredoxin-interacting protein, miRNA: micro RNA, ATP: adenosine triphosphate, ROS: reactive oxygen species, P2rx7: purinergic P2X7 receptor, iNKT: invariant natural killer T cell, TNFα: tumor necrosis factor α. The red symbol illustrates inhibition of uric acid by allopurinol. The arrow means that activation of TLR signaling induces expression of NLRP3 and IL-1b mRNA in the nucleus.

Extracellular ATP and uric acid were identified as DAMPs elevated in alcohol-induced liver injury [77]. Both were released from alcohol-damaged hepatocytes and stimulated IL-1β production in liver immune cells [76,77]. Interestingly, pharmacological inhibition of uric acid synthesis with allopurinol or treatment with probenecid, which depletes uric acid and blocks ATP-induced P2rx7 signaling, improved pathology, and therefore might be potential therapeutic strategies for the treatment of ALD [77]. Gentiopicroside, a substance with hepatoprotective properties, also displayed beneficial effects on ALD severity, which was attributed to inhibition of P2rx7-mediated NLRP3 inflammasome activation [97]. The miRNA miR-148a was identified as an inhibitor of NLRP3 inflammasome activation in ALD. Levels of miR-148a were substantially decreased in livers of patients with ALD and in alcohol-fed mice. As miR-148a is a suppressor of the thioredoxin-interacting protein (TXNIP), this led to TXNIP overexpression in hepatocytes facilitating their pyroptosis. Moreover, lack of miR-148a-mediated suppression of TXNIP favored its association with NLRP3 and activated the inflammasome pathway. Conclusively, miR-148a delivery to hepatocytes was shown to prevent inflammasome activation and attenuated ALD pathogenesis [78] (Figure 1).

### 4.3. Drug-Induced Liver Injury

A variety of studies have indicated that the inflammation-induced aggravation of APAP-induced liver injury is driven by hepatic infiltration of neutrophils and inflammatory monocytes [110,111,112,113] mediated by release of DAMPs [114,115,116]. Extracellular ATP was shown to function as DAMP in APAP-induced pathology that induced IL-1β expression in Kupffer cells and aggravated liver injury. Inhibition of extracellular ATP signaling by using the P2rx7 antagonist A438079 or by administration of soluble CD39 markedly reduced APAP-induced necrosis and mortality demonstrating the importance of this DAMP for APAP pathology [79]. Furthermore, in APAP-induced liver injury, apoptotic hepatocytes were shown to release free DNA, which in turn activated TLR9 expressed by LSEC [80]. Usually, TLR9 recognizes bacterial DNA, however, it was found that TLR9 is also activated by mammalian DNA that was modified by DNase-mediated cleavage and aberrant methylation and oxidative damage [117,118]. Free DNA-mediated TLR9 signaling in LSEC induced NLRP3 inflammasome activation, IL-1β production, and subsequent neutrophil infiltration [80]. The relevance of TLR9 as potential therapeutic target in APAP-induced liver injury was illustrated by two studies demonstrating that lack of TLR9 signaling ameliorated tissue damage [80,98]. Direct targeting of the NLRP3 inflammasome was also proposed as therapeutic option. Aspirin and benzyl alcohol were protective in APAP-induced pathology and it has been suggested that this was mediated through inhibition of NLRP3 inflammasome activation and neutrophil infiltration [80,98].

Thus, the NLRP3 inflammasome has been identified as a driver of sterile inflammation in APAP-induced liver injury by inducing hepatic neutrophil recruitment. However, the role of the NLRP3 inflammasome and neutrophils in APAP-induced liver injury have been controversially discussed in the literature. Several studies could not support the assumption that neutrophils aggravate APAP-induced liver injury [119,120,121]. It was further shown that lack of P2rx7 or IL-1R did not ameliorate disease pathogenesis and also treatment with IL-1β or the pan-caspase inhibitor Z-VD-fmk had no effects on liver damage indicating that ATP and IL-1β have no inflammatory function in APAP-induced liver injury [81,82,122]. The role of the NLRP3 inflammasome in APAP-induced liver damage is discussed in detail by Woolbright et al. [123] and it becomes quite obvious that further work is needed to establish whether the inflammasome pathway might be a therapeutic target in this condition. 

### 4.4. Liver I/R

Production of ROS by Kupffer cells has been proposed as a critical factor for sterile inflammation in liver I/R injury [124]. ROS function as DAMP and activate the NLRP3 inflammasome by promoting association of TXNIP with NLRP3 [89] thereby linking oxidative stress with inflammation. Inhibition of Kupffer cells by gadolinium chloride and administration of the ROS inhibitor *N*-acetylcysteine attenuated liver injury and IL-1β production supporting the critical role of Kupffer cells and ROS in hepatic I/R [83]. Extracellular histones were identified as new DAMPs that activated the NLRP3 inflammasome in Kupffer cells through TLR9-dependent generation of ROS, which in turn aggravated hepatic damage by recruitment of neutrophils and inflammatory monocytes [84]. Further, HMGB1 is another DAMP that was found to trigger hepatic I/R injury [85].

Up-regulation of the NLRP3 inflammasome and IL-1β expression in hepatic I/R injury have been shown in a variety of studies [83,84,86,87,88], and lack of NLRP3 or caspase-1 [84] or blockage of IL-1β with an anti-IL-1β antibody [85] improved disease pathology further reinforcing the importance of the NLRP3 inflammasome for liver I/R pathogenesis. The findings in *Casp1*^−/−^ mice were not confirmed by another study and the authors suggested a caspase-1-independent function of NLRP3 and proposed that NLRP3 is involved in the regulation of neutrophil function [87]. However, gene silencing of NLRP3 by hydrodynamic tail vein injection of a pNALP3shRNA ameliorated disease pathology associated with reduced caspase-1 activation, IL-1β production and neutrophil infiltration [86], strongly indicating that NLRP3 inflammasome activation aggravates liver I/R injury.

The heat shock transcription factor 1 (HSF1) was identified as a regulator of NLRP3 inflammasome activation in liver I/R. HSF1 is induced in response to oxidative stress and facilitates cell survival [125]. Mice with a myeloid-specific HSF1 deletion displayed exaggerated liver I/R damage, increased NLRP3 and IL-1β expression, and neutrophil recruitment. Lack of myeloid HSF1 also reduced β-catenin levels but enhanced expression of the transcription factor X-box-binding protein (XBP1), which is an activator of the NLRP3 inflammasome. In the proposed mechanism, HSF1 induces β-catenin expression and the HSF1/β-catenin axis suppresses expression of XBP1 and subsequent NLRP3 inflammasome activation in liver I/R injury [90].

### 4.5. AIH

Concanavalin (Con)A-induced hepatitis is a well accepted murine model of immune-mediated liver injury, reflecting several immune mechanisms responsible for liver pathology of acute autoimmune hepatitis (AIH). ConA-induced hepatitis is driven by T-cell and macrophage activation, massive pro-inflammatory cytokines production and hepatocyte death thereby mimicking pathogenic features of AIH [126,127,128]. Recent studies have indicated that the NLRP3 inflammasome drives pathogenesis of ConA-induced hepatitis. These studies illustrated that liver pathology was accompanied by production of ROS [91,92] and elevated levels of NLRP3, IL-1β, caspase-1 and pyroptosis-mediated cell death [93]. Lack of NLRP3 or caspase-1 ameliorated liver injury and was associated with reduced IL-1β production [93,94]. Moreover, ConA was shown to induce NLRP3 inflammasome and caspase-1 activation and IL-1β production in macrophages in vitro [94]. The critical role of IL-1β/NLRP3 in the pathogenesis of ConA-induced hepatitis was also supported by an intervention study using an IL-1R antagonist that suppressed hepatic inflammation by diminishing ROS production and NLRP3 inflammasome activation [93]. IL-1β also appears to be involved in the pathology of human AIH since IL-1β levels were elevated in AIH patients and correlated with aggravation of hepatitis [31]. So far, there is no evidence for extracellular ATP-mediated activation of the NLRP3 inflammasome in AIH. However, one study, which did not link P2rx7 signaling with inflammasome activation, reported that *P2rx7*^−/−^ mice were markedly protected from liver damage in ConA-induced hepatitis [129]. Based on the findings in other sterile inflammation-associated liver diseases, one might speculate that the suppressive effect is due to impaired NLRP3 inflammasome activation in the absence of P2rx7.

Regulation of NLRP3 gene expression by miRNAs has been suggested as a mechanism involved in immune regulation in AIH. The miRNA miR-223 negatively regulates NLRP3 expression [130], and in a murine AIH model induced by hepatic S100 injection, application of exosomes containing miR-223 decreased NLRP3 and caspase-1 expression and attenuated liver injury [95].

## 5. The Alarmin IL-33

### 5.1. Molecular Biology

IL-33 is a member of the IL-1 family of cytokines and has been identified as the only ligand for suppression of tumorigenicity 2 (ST2) receptor that belongs to the IL-1 receptor family [131]. IL-33 is a nuclear cytokine, which is released after cell death as a consequence of severe tissue injury. Upon synthesis, cytosolic IL-33 translocates to the nucleus where it associates with chromatin. The N-terminal domain of IL-33 contains a nuclear localization sequence and a chromatin-binding domain and is essential for its nuclear translocation [132]. So far, passive release from necrotic cells has been reported as main mechanism for appearance of extracellular IL-33. There are two forms of IL-33: full-length IL-33 (pro-IL-33) and mature IL-33. Pro-IL-33 is located in the nucleus but its nuclear function is not clear [133]. Since deletion of the N-terminal domain leads to sustained IL-33 release and lethal inflammation [134], nuclear binding of IL-33 to chromatin has been suggested as a mechanism to restrict its pro-inflammatory activity in homeostasis. Upon release, pro-IL-33 is often cleaved by serine proteases into mature IL-33, which has a higher biological activity than pro-IL-33 and cannot translocate into the nucleus [135].

### 5.2. The IL-33 Receptor ST2

Both IL-33 forms mediate cytokine function by binding to their receptor ST2 that forms a heterodimeric complex with the IL-1R accessory protein. Signaling via ST2 activates the MyD88 and NFκB pathways subsequently inducing cell activation, differentiation, and survival as well as expression of cytokines and chemokines [136,137]. ST2 has two main splice variants dependent on the promotor being used: the transmembrane form ST2 and the soluble form sST2. While ST2 mediates IL-33 signaling, sST2 acts as a decoy receptor for extracellular IL-33 thereby restricting its biological activity after release [138].

### 5.3. IL-33 Expression and Immune Cell Regulation

IL-33 is constitutively expressed in the nuclei of human endothelial cells, epithelial cells, keratinocytes, fibroblast, smooth muscle cells, and glial cells [139]. In mice, epithelial cells rather than endothelial cells, and also hepatocytes constitutively express IL-33. However, during inflammation, IL-33 expression is induced in a variety of cell types including endothelial cells [140,141,142]. 

It has been proposed that IL-33 acts as an “alarmin” released in response to cell damage and necrosis to alert the immune system [143]. IL-33 stimulates activation of ST2-expressing lymphoid and myeloid cells such as Th2 cells, type 2 innate lymphoid cells (ILC2), regulatory T cells (Tregs), NKT cells, CD8^+^ T cells, M2 macrophages, neutrophils, eosinophils, basophils, mast cells, and NK cells [144]. IL-33 has been initially identified as an inducer of Th2-cell differentiation and type 2 cytokine production [131]. Beyond that, IL-33 induces activation and expansion of a Treg subset expressing ST2 that predominantly localizes in non-lymphoid organs. IL-33/ST2 signaling further ensures proper Treg function during inflammation and Treg adaption to the inflammatory environment [145,146,147]. IL-33 also activates ILC2, a lymphoid cell population of the innate immune system, to express the type 2 cytokines IL-5 and IL-13 and the epidermal growth factor amphiregulin (AREG), by which they contribute to tissue regeneration upon injury [148,149,150]. Moreover, IL-33 facilitates macrophage polarization into an alternatively activated M2 phenotype [151], and promotes DC-mediated Treg expansion by inducing IL-2 expression in DCs [152].

### 5.4. IL-33 in Inflammatory Diseases

IL-33 is a pleiotropic cytokine, which is well illustrated in the diverse range of diseases in whose pathologies IL-33 exert very different functions. A pathogenic role of IL-33 has been described in airway diseases. In patients with asthma, IL-33 levels were elevated and correlated with disease severity [153]. IL-33 promoted airway hyperreactivity in murine influenza virus infection by activation of IL-13-expressing lung ILC2 [154], and induced airway inflammation by driving recruitment of eosinophils [155] and M2 macrophage polarization [151]. Also, in patients with rheumatoid arthritis, higher levels of IL-33 were correlated with more severe disease [156], and blockage of IL-33/ST2 signaling ameliorated murine collagen-induced arthritis associated with decreased IFNγ production [157]. In inflammatory bowel disease (IBD), divergent functions of IL-33 have been depicted. On the one hand, mucosal IL-33 levels correlated with disease severity in IBD patients and in mice with experimental colitis [158], and lack of IL-33 or ST2 attenuated dextran sodium sulfate-induced colitis by protection against IL-33-mediated epithelial damage [159] and enhanced wound healing response [160]. On the other hand, administration of IL-33 protected mice in experimental colitis through expansion of Tregs [161], and induction of AREG expression in gut ILC2 [149]. In organ transplantation, IL-33 treatment prolonged murine cardiac allograft survival by expansion of immunosuppressive myeloid-derived suppressor cells and intragraft Tregs [162]. IL-33 can also induce different immune responses dependent on the treatment regime. In acute graft-versus-host disease after allogeneic hematopoietic cell transplantation, IL-33 administered during the peak of the inflammatory response increased type 1 cytokine expression and aggravated mortality [163] whereas IL-33 treatment before body irradiation expanded ST2^+^ Tregs and accelerated acute lethality [164].

**Figure 2:**
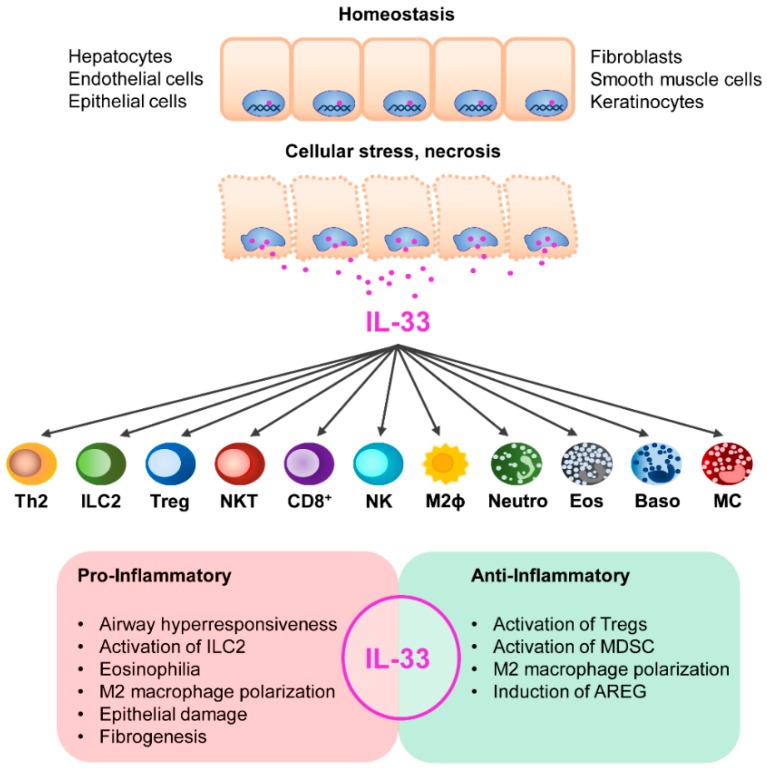
Immune regulatory function of IL-33 in inflammatory diseases. In homeostasis, IL-33 is located in the nucleus where it is associated with chromatin. IL-33 is released from necrotic cells and activates a variety of lymphoid and myeloid immune cells expressing the IL-33 receptor ST2. Dependent on the disease, IL-33 exerts very different functions. On the one hand, IL-33 can drive inflammation while on the other hand it also supports immunosuppression. ILC2: type 2 innate lymphoid cell, Treg: regulatory T cell, NKT: natural killer T cell, NK: natural killer cell, M2ϕ: M2 macrophage, Neutro: neutrophil, Eos: eosinophils, Baso: basophil. MC: mast cell, MDSC: myeloid-derived suppressor cell, AREG: amphiregulin.

Thus, IL-33 exerts pro- and anti-inflammatory function dependent on the disease. On the one hand, IL-33 drives immunity by promoting type 2 or type 1 immune responses, eosinophilia, ILC2 activation, M2 macrophage polarization, and epithelial cell damage whereas on the other hand, IL-33 suppresses inflammation by expansion of Tregs and activation of myeloid-derived suppressor cells. IL-33 can further facilitate tissue repair by inducing AREG expression in ILC2. It is worth noting that by these mechanisms, IL-33 may also be involved in pathological processes of tissue regeneration, fibrosis, and carcinogenesis in chronic inflammation (Figure 2).

## 6. IL-33/ST2 Axis in Sterile Inflammation-Associated Liver Diseases

There is some evidence that IL-33 is involved in the pathology of liver diseases. In general, IL-33 has been proven as a pathogenic marker in patients with acute liver failure and acute-on-chronic liver failure [165], liver cirrhosis [166] as well as chronic hepatitis B virus infection [167] or hepatitis C virus infection [168]. IL-33 has been further suggested as pro-fibrotic factor that is associated with liver fibrosis in chronic liver disease in mice and humans [169]. The immunoregulatory function of IL-33 in liver inflammation is still under investigation and in the next section, we will summarize the current studies regarding the role of the IL-33/ST2 axis in sterile liver inflammation and provide an overview in Table 3.

### 6.1. NAFLD

There are conflicting data about the role of IL-33 in the pathogenesis of NAFLD. In NASH, predominantly endothelial cells, and HSC up-regulate expression of nuclear IL-33 [170]. Elevated IL-33 and ST2 levels were determined in NASH patients [170] and mice with diet-induced steatohepatitis [170,171,172]. However, the contribution of IL-33 to disease pathology remains less clear since lack of endogenous IL-33 did neither affect fibrosis nor immune cell composition in the liver [172]. In contrast, exogenous IL-33 induced opposing effects on disease progression as steatosis was attenuated while hepatic fibrosis was exacerbated. The beneficial effect of exogenous IL-33 on steatosis was attributed to a shift from an inflammatory Th1 response to an anti-inflammatory Th2 response. It was suggested that the concurrent pro-fibrotic effect of IL-33 might be mediated by induction of IL-13 expression in ILC2 [170] as shown in murine models of hepatic fibrosis, in which ILC2-derived IL-13 activated HSC, the key drivers of hepatic fibrosis [166]. The pro-fibrotic effect of exogenous IL-33 in NASH pathology was confirmed by another study and correlated with an increased frequency of IL-13-expressing monocytes/macrophages. The β-galactosidase-binding lectin galectin-3 was identified as a regulator of the IL-33/ST2 pathway in NASH since it enabled IL-33-induced ST2 expression in macrophages as a prerequisite for IL-13 production [173]. The divergent effects of exogenous and endogenous IL-33 in NASH have been discussed and were attributed to unphysiological systemic IL-33 levels after IL-33 treatment, which might affect other immune cells than locally released IL-33, differences in the used mouse models, and a potential suppressive effect of nuclear pro-IL-33. The authors hypothesized that pro-IL-33 suppresses inflammatory cytokine expression that might compensate the pro-fibrotic effect of IL-33 released during NASH. In contrast, administration of high concentrations of exogenous IL-33 might overcome the inhibitory effect of pro-IL-33 leading to aggravated fibrosis in diet-induced steatohepatitis [172].

### 6.2. ALD

So far, there is no direct evidence that IL-33 plays a role in ALD. In one study, IL-33 and sST2 levels were compared in patients with different stages of ALD and were only enhanced in patients with severe decompensated ALD but not in patients with compensated ALD or heavy drinkers [174]. In mice, hepatic IL-33 expression was increased in alcohol-fed mice [47] whereas serum levels of IL-33 and sST2 were not elevated and lack of IL-33 did not affect alcohol-induced liver injury. Low hepatic cell death in the currently available models of ALD has been suggested as reason for the missing effect of IL-33 on disease pathology [174] fitting to the human data where only patients with the severest form of ALD showed elevated IL-33 levels.

Interestingly, an IL-33-independent function of ST2 has been proposed in ALD. While lack of IL-33 did not alter pathology, ST2 deficiency aggravated alcohol-induced steatosis and liver damage associated with NFκB activation and inflammatory cytokine expression in hepatic macrophages. In mild alcohol-induced liver injury without substantial cell death, ST2 was suggested as negative regulator of NFκB in macrophages thereby limiting their inflammatory activity [174].

### 6.3. Drug-Induced Liver Injury

The knowledge about the function of IL-33 in drug-induced liver injury is very limited yet. One study reported that in APAP-induced liver injury, formation of massive necrosis was associated with the release of IL-33 by hepatocytes and neutrophil infiltration. Lack of ST2 did not impair initial liver damage but prevented progression to extensive necrosis and it was concluded that IL-33 amplifies liver damage by neutrophil recruitment [175].

### 6.4. Liver I/R

It is well known that neutrophils are critical for the pathology of hepatic I/R injury. Neutrophils were shown to exacerbate liver I/R injury by IL-33-induced formation of neutrophil extracellular traps (NET) in the liver sinusoids that induced hepatocyte death and Kupffer cell activation. Mechanistically, LSEC released IL-33 during hepatic I/R injury that induced NET formation in ST2-expressing neutrophils [176,186]. Administration of IL-33 immediately after liver I/R increased NET formation and liver injury [176] whereas IL-33 pre-treatment reduced neutrophil infiltration and hepatocyte death by inducing expression of the anti-apoptotic protein Bcl-2 [177].

A potential beneficial effect of sST2 on liver I/R pathology was tested by using an expression plasmid coding for a murine soluble ST2-human Fc fusion protein (sST2-Fc). Overexpression of sST2-Fc reduced liver I/R injury and the authors proposed that sST2-Fc prevented TLR4-mediated Kupffer cell activation and subsequent pro-inflammatory cytokine production [178]. A decoy function of sST2 for IL-33 released during hepatic I/R injury was not addressed in this study but this might be an interesting point since IL-33 levels were increased in patients undergoing liver resection [176] and in mice after liver I/R [176,177].

### 6.5. AIH

Divergent effects of the IL-33/ST2 axis have been described in ConA-induced hepatitis. NKT cells were shown to induce strong nuclear IL-33 expression in hepatocytes during ConA-induced liver injury [180] that was regulated by TNF-related apoptosis-inducing ligand (TRAIL) [181]. We have recently demonstrated that hepatic release of IL-33 was associated with formation of large necrotic lesions during ConA-induced hepatitis and aggravation of disease pathology by activation of hepatic ILC2s expressing IL-5 and IL-13. This was associated with infiltration of inflammatory eosinophils. Therefore, we suggested that ILC2-derived IL-5 contribute to hepatic eosinophil activation and recruitment thereby promoting liver inflammation and tissue damage [182]. The findings that blockage of IL-33 signaling through an anti-IL-33 antibody reduced NKT-cell activation and ameliorated disease pathogenesis and that pre-treatment with sST2-Fc attenuated liver injury [183] further supported the pro-inflammatory role of IL-33 in ConA-induced hepatitis. A beneficial effect of anti-IL-33 antibody treatment on disease pathology was also demonstrated in an experimental AIH model induced by injection of S-100 antigen. Moreover, in patients with AIH, increased serum levels of IL-33 were positively correlated with liver injury and inflammatory cytokine production and both IL-33 and sST2 levels decreased after immunosuppression therapy [179].

**Figure 3:**
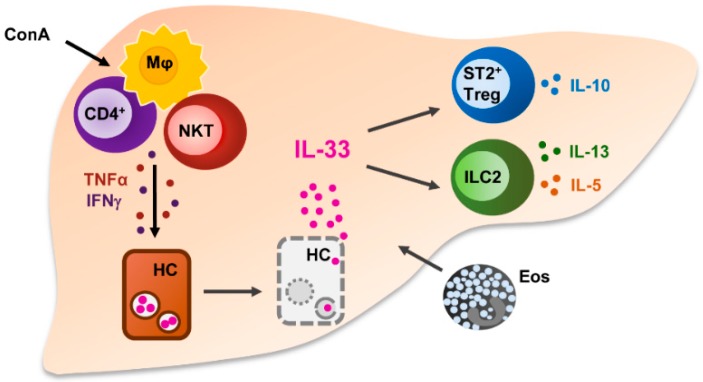
Immune regulatory function of IL-33 in ConA-induced hepatitis. In homeostasis, IL-33 is located in the nucleus of hepatocytes. During ConA-induced hepatitis, activated NKT cells, CD4^+^ T cells and macrophages produce the inflammatory cytokines TNFα and IFNγ subsequently leading to hepatocyte death by necrosis and release IL-33 in liver tissue. Extracellular IL-33 activates ILC2, which is associated with hepatic infiltration of inflammatory eosinophils. IL-33 also activates ST2^+^ Tregs that might exert immunosuppressive function in sterile liver inflammation. ConA: concanavalin A, Mϕ: macrophage, NKT: natural killer T cell, TNFα: tumor necrosis factor α, IFNγ: interferon γ, HC: hepatocyte, ST2: suppression of tumorigenicity 2, Treg: regulatory T cell, ILC2: type 2 innate lymphoid cells, Eos: eosinophils.

In contrast to these studies, a protective effect of IL-33 and ST2 in ConA-induced hepatitis was also described. Lack of ST2 aggravated liver injury and increased inflammatory cytokine expression while the number of Tregs was reduced [184]. IL-33 deficiency also rendered mice more susceptible to ConA-induced liver injury [181,185] and this was addressed to increased NK cell infiltration together with a reduced frequency of Tregs expressing ST2 [185]. The assumption that ST2^+^ Tregs contribute to immune regulation in ConA-induced hepatitis was promoted by another study showing an enhanced frequency of this Treg subset in the inflamed liver. Moreover, administration of IL-33 before induction of hepatitis expanded hepatic ST2^+^ Tregs and potently suppressed liver injury [182]. The protective effect of IL-33 pre-treatment on disease pathology was reproduced by another study [184] while administration of IL-33 together with ConA exacerbated liver injury and necrosis [183]. Since ST2^+^ Tregs have been described as an activated Treg subset with potent immunosuppressive function in inflammation [145,146,147], it could be speculated that absence of ST2^+^ Tregs in ST2-deficient mice and defective activation of this Tregs subset in IL-33-deficient mice might be responsible for the observed aggravated liver injury in ConA-induced hepatitis (Figure 3).

## 7. NLRP3 Inflammasome and IL-33 in Sterile Inflammation-Associated Liver Diseases

Initially, the NLRP3 inflammasome pathway has been proposed to be involved in the maturation of IL-33. IL1β and IL-33 are members of the IL-1 family of cytokines and both are intracellularly produced as pro-cytokines [131]. Similar to IL-1β, cleavage of pro-IL-33 into mature IL-33 by caspase-1 was indicated to be essential for optimal biologic activity [131]. However, this finding was not supported by other studies. In contrast, it was reported that pro-IL-33 is biologically active and that caspase-1 processing resulted in inactivation rather than activation of IL-33 [187]. Another study found no evidence that IL-33 is a physiological substrate for caspase-1 and instead demonstrated efficient processing by the apoptosis-associated caspase-3 and -7 that attenuated the biological activity of IL-33 [188], whereas serine proteases were shown to process IL-33 into mature forms with increased biological activity [135]. Moreover, caspase-1-deficient macrophages were able to release IL-33 after LPS stimulation [189] strongly indicating that the NLRP3 inflammasome and caspase-1 are dispensable for IL-33 maturation.

It appears that both the NLRP3 inflammasome and the IL-33 pathway can become activated in sterile inflammation-associated liver diseases. Whether these pathways act synergistically or antagonistically in sterile liver inflammation is still largely unknown. One study suggests a link between NLRP3 inflammasome and IL-33 signaling in diet-induced steatohepatitis. The authors proposed that NLRP3 inflammasome-induced IL-1β expression by macrophages contributed to necrotic hepatocyte death and release of IL-33, which in turn induced expression of the pro-fibrotic cytokine IL-13 in macrophages that might promote HSC activation, fibrogenesis, and progression of NASH [173]. In ConA-induced hepatitis, lack of IL-33 was associated with increased hepatic IL-1β expression and more severe liver injury, however, a possible link between the NLRP3 inflammasome and IL-33 was not analyzed [185]. In contrast, other studies found no interaction between both pathways. Alcohol-fed mice showed up-regulation of both NLRP3/caspase-1 and pro-IL-33 in the liver. However, since comparable levels of cleaved hepatic IL-33 protein were found in Casp1^−/−^ mice, it was suggested that cleavage of IL-33 occurred independently of NLRP3/caspase-1 in alcohol-induced liver injury [47], fitting to the studies mentioned above. Further, in a murine model of acute viral hepatitis, Il33^−/−^ mice showed no altered expression of IL-1β [190].

Despite the weak evidence for an interaction of the NLRP3 inflammasome and IL-33 pathway in sterile liver inflammation, a NLRP3-IL-33 axis has been shown in other diseases. In house dust mite-induced allergic lung inflammation, a regulatory role of caspase-1 on IL-33 release has been proposed. The authors found that caspase-1 restrained expression of cleaved IL-33 and that up-regulation of airway inflammation in Casp1^−/−^ mice correlated with higher bioactive IL-33 expression and increased lung infiltration of eosinophils [191]. In contrast to this study, lack of caspase-1 reduced eosinophilia and mature bioactive lung IL-33 levels in an asthma exerbation model [192]. Another study showed that IL-33 treatment reduced NLRP3 inflammasome activation and IL-1β production in microglia and intracerebral monocytes in experimental cerebral malaria thereby ameliorating pathology in the brain [193]. In helminth infection, NLRP3 inflammasome activation and IL-1β production in the intestine were identified as mechanism to prevent parasite rejection and to establish chronic infection by suppressing expression of IL-33 [194]. It was further shown in vitro that IL-33 can induce IL-1β production by bone marrow-derived mast cells [195]. Based on these findings and the fact that both the NLRP3 inflammasome and IL-33 pathway play a role in the pathology of most of the sterile inflammation-associated liver diseases, it would make sense to study a possible NLRP3-IL-33 axis in sterile liver inflammation in more detail.

## 8. Conclusions

There is compelling evidence that activation of the NLRP3 inflammasome leads to progression of sterile inflammation-associated liver diseases and therefore, components of this inflammatory pathway represent promising new therapeutic targets. IL-33 has been proven as a pathogenic factor in chronic liver disorders, however, its particular role in sterile liver inflammation is not yet fully understood. It appears that IL-33 drives sterile liver inflammation by mediating activation of ILC2, Kupffer cells, neutrophils, and eosinophils while on the other hand, IL-33 also suppresses disease severity by promoting Treg function. Thus, targeting IL-33 (e.g., by using an anti-IL-33 antibody) must be well considered and is dependent on the main pathway triggered by IL-33 in a given liver disease. The situation is further complicated by the finding that treatment with recombinant IL-33 as therapeutic option in sterile liver inflammation often results in different outcomes dependent on time point and duration of IL-33 administration. However, further studies are required to elucidate whether targeting of the IL-33/ST2 axis might be a novel therapeutic strategy for treatment of sterile inflammation-related liver disease.

## Figures and Tables

**Table 1:** List of inflammasome components in sterile liver disease.

Disease	Inflammasome Components	References
**NAFLD**	Elevated NLRP3 and IL-1β levels correlated with liver fibrosis in NASH patients and were increased in different murine models of diet-induced steatohepatitis	[62,63,64,65,66]
	*Nlrp3^−/−^* mice showed marked protection from diet-induced liver injury whereas disease pathology was accelerated in *Nlrp3* knock-in mice	[62,64,67]
	Caspase-1 activation in Kupffer cells triggered progression of diet-induced steatohepatitis	[68,69]
	Extracellular ATP activated the NLRP3 inflammasome in diet-induced steatohepatitis and *P2rx7^−/−^* mice showed reduced liver fibrosis	[70]
	Cholesterol crystals were found in steatotic hepatocytes of patients and mice with NASH, which induced NLRP3 and caspase-1 activation in Kupffer cells	[48,71,72]
**ALD**	Elevated serum IL-1β levels were shown in patients with ALD	[73,74]
	NLRP3/caspase-1-mediated IL-1β production by Kupffer cells aggravated murine ALD	[47]
	Kupffer cell-derived IL-1β activated invariant NKT cells, which in turn promoted alcohol-induced liver injury by recruiting neutrophils	[75]
	Extracellular ATP and uric acid were released from alcohol-damaged hepatocytes and stimulated IL-1β production in liver immune cells	[76,77]
	The miRNA miR-148a inhibited NLRP3 inflammasome activation in alcohol-fed mice and levels of hepatic miR-148a were reduced in patients and mice with ALD	[78]
**Drug-Induced Liver Injury**	Extracellular ATP induced IL-1β production in Kupffer cells and aggravated murine APAP-induced liver injury	[79]
	The P2rx7 antagonist A438079 and soluble CD39 reduced APAP-induced necrosis	[79]
	Free DNA was released by apoptotic hepatocytes, which induced NLRP3 inflammasome activation in LSEC and neutrophil recruitment in APAP-induced liver injury	[80]
	Lack of P2rx7, IL-1R and treatment with IL-1β or the pan-caspase inhibitor Z-VD-fmk did not alter pathology of APAP-induced liver injury	[81,82]
**Liver I/R**	Elevated NLRP3 and IL-1β levels were shown in liver I/R injury in a variety of studies and *Nlrp3^−/−^* and *Casp-1^−/−^* mice showed improved disease pathology	[83,84,85,86,87,88]
	ROS activated the NLRP3 inflammasome in Kupffer cells in liver I/R injury by promoting association of TXNIP with NLRP3	[83,89]
	Extracellular histones activated the NLRP3 inflammasome in Kupffer cells in liver I/R injury through generation of ROS leading to recruitment of neutrophils and monocytes	[84]
	HMGB1 triggered hepatic I/R injury by activation of the NLRP3 inflammasome	[85]
	HSF1 regulated NLRP3 inflammasome activation in liver I/R injury by suppressing expression of XBP1, an activator of the NLRP3 inflammasome	[90]
**AIH**	Elevated IL-1β levels in patients with AIH were correlated with aggravation of hepatitis	[31]
	Elevated levels of NLRP3, IL-1β, caspase-1, ROS production and pyroptosis-mediated cell death were shown in murine ConA-induced hepatitis	[91,92,93,94]
	ConA induced NLRP3 inflammasome activation and IL-1β production in macrophages	[94]
	An IL-1R antagonist suppressed ConA-induced hepatitis by diminishing ROS production and NLRP3 inflammasome activation	[93]
	The miRNA miR-223 negatively regulated NLRP3 expression and attenuated liver injury in an experimental AIH model	[95]

**Table 2:** Potential therapeutic strategies targeting the NLRP3 inflammasome pathway in sterile liver disease.

Disease	Therapeutic Strategy	References
**NAFLD**	The cholesterol-lowering drug ezetimibe reduced cholesterol crystal formation and fibrosis in murine diet-induced steatohepatitis	[96]
	The small molecule NLRP3 inhibitor MCC950 inhibited Kupffer cell activation and attenuated inflammation and fibrosis in murine diet-induced steatohepatitis	[48,72]
**ALD**	Inhibition of uric acid synthesis with allopurinol and treatment with probenecid, which depletes uric acid and blocks ATP-induced P2rx7 signaling, improved pathology of murine alcohol-induced liver injury	[77]
	The hepatoprotective substance Gentiopicroside inhibited P2rx7-mediated NLRP3 inflammasome activation and ameliorated pathology of murine ALD	[97]
**Drug-Induced Liver Injury**	The P2rx7 antagonist A438079 or soluble CD39 inhibited extracellular ATP signaling and reduced murine APAP-induced liver injury and mortality	[79]
	Aspirin and benzyl alcohol were protective in APAP-induced pathology probably by inhibiting the NLRP3 inflammasome activation and neutrophil infiltration	[80,98]
**Liver I/R**	The ROS inhibitor *N*-acetylcysteine inhibited NLRP3 inflammasome activation and attenuated murine liver I/R injury	[83]
	Blockage of IL-1β signaling by an anti-IL-1β antibody improved disease pathology of murine liver I/R injury	[85]
**AIH**	An IL-1R antagonist diminished ROS production and NLRP3 inflammasome activation and suppressed liver inflammation in murine ConA-induced hepatitis	[93]

**Table 3:** Role of IL-33/ST2 in sterile liver diseases.

Disease	Role of IL-33/ST2	References
**NAFLD**	Serum IL-33 and ST2 levels were elevated in patients with NASH and mice with diet-induced steatohepatitis	[170,171,172]
	*Il33^−/−^* mice showed no altered pathology of diet-induced steatohepatitis	[172]
	IL-33 treatment attenuated steatosis but aggravated fibrosis in diet-induced steatohepatitis	[170]
	Galectin-3 regulated IL-33-induced expression of the pro-fibrotic cytokine IL-13 in hepatic macrophages in diet-induced steatohepatitis	[173]
**ALD**	Serum IL-33 and sST2 levels were elevated in patients with severe decompensated ALD but not in patients with compensated ALD and heavy drinkers	[174]
	Serum levels of IL-33 were not elevated in alcohol-fed mice and lack of IL-33 did not affect pathogenesis of alcohol-induced liver injury	[174]
	Hepatic IL-33 expression was increased in murine alcohol-induced liver injury	[47]
**Drug-Induced Liver Injury**	Hepatocytes released IL-33 that induced neutrophil infiltration in murine APAP-induced liver injury	[175]
	*Il1rl1^−/−^* mice developed initial liver damage but were protected against progression to extensive necrosis in APAP-induced liver	[175]
**Liver I/R**	LSEC released IL-33 in murine hepatic I/R injury that induced formation of NETs by neutrophils, which promoted hepatocyte death and Kupffer cell activation	[176]
	IL-33 treatment after liver I/R increased NET formation and liver injury	[176]
	IL-33 treatment before liver I/R reduced hepatocyte death by induction of Bcl-2	[177]
	sST2-Fc attenuated hepatic I/R injury by inhibiting Kupffer cell activation	[178]
**AIH**	Elevated serum IL-33 levels correlated with liver injury in AIH patients and both IL-33 and sST2 levels were decreased after immunosuppression therapy	[179]
	NKT cells induced IL-33 expression in hepatocytes in murine ConA-induced hepatitis	[180]
	TRAIL regulated IL-33 expression in hepatocytes during ConA-induced hepatitis	[181]
	IL-33 aggravated ConA-induced liver injury by activation of hepatic ILC2	[182]
	Blockage of IL-33 attenuated liver injury in two experimental models of AIH	[179,183]
	*Il1rl1^−/−^* mice showed aggravated ConA-induced liver injury and reduced Treg numbers	[184]
	*Il33^−/−^* mice showed exaggerated pathology of ConA-induced hepatitis, increased NK cell infiltration and reduced frequency of ST2^+^ Tregs	[181,185]
	IL-33 pre-treatment expanded hepatic ST2^+^ Tregs and suppressed ConA-induced hepatitis	[182,184]
	IL-33 treatment together with ConA challenge aggravated ConA-induced hepatitis	[183]

## Abbreviations

NASH	nonalcoholic steatohepatitis
I/R	ischemia reperfusion
AIH	autoimmune hepatitis
DAMPs	damage-associated molecular patterns
IL	interleukin
NAFLD	nonalcoholic fatty liver
ALD	alcoholic liver disease
LPS	lipopolysaccharide
APAP	acetaminophen
ROS	reactive oxygen species
Treg	regulatory T cells
PAMPs	pathogen-associated molecular pattern
TLR	Toll-like receptor
TNFα	tumor necrosis factor α
ATP	adenosine triphosphate
HMGB1	high mobility group box 1
NLR	nucleotide-binding oligomerization domain-like receptor
IL-1R	IL-1 receptor
HSC	hepatic stellate cells
TXNIP	transcription factor X-box-binding protein
ConA	Concanavalin A
ST2	suppression of tumorigenicity 2
ILC2	type 2 innate lymphoid cells
AREG	amphiregulin
IBD	inflammatory bowel disease
NET	neutrophil extracellular traps

## References

[1] Rock K.L., Latz E., Ontiveros F., Kono H. (2010). The Sterile Inflammatory Response. Annu. Rev. Immunol..

[2] Kubes P., Mehal W.Z. (2012). Sterile inflammation in the liver. Gastroenterology.

[3] Cohen J.C., Horton J.D., Hobbs H.H. (2011). Human fatty liver disease: Old questions and new insights. Science.

[4] Roden M. (2006). Mechanisms of Disease: Hepatic steatosis in type 2 diabetes—Pathogenesis and clinical relevance. Nat. Clin. Pract. Endocrinol. Metab..

[5] Wree A., Broderick L., Canbay A., Hoffman H.M., Feldstein A.E. (2013). From NAFLD to NASH to cirrhosis-new insights into disease mechanisms. Nat. Rev. Gastroenterol. Hepatol..

[6] Starley B.Q., Calcagno C.J., Harrison S.A. (2010). Nonalcoholic fatty liver disease and hepatocellular carcinoma: A weighty connection. Hepatology.

[7] Day C.P., James O.F.W. (1998). Steatohepatitis: A tale of two “Hits”?. Gastroenterology.

[8] Gentile C.L., Pagliassotti M.J. (2008). The role of fatty acids in the development and progression of nonalcoholic fatty liver disease. J. Nutr. Biochem..

[9] Sanyal A.J., Campbell-Sargent C., Mirshahi F., Rizzo W.B., Contos M.J., Sterling R.K., Luketic V.A., Shiffman M.L., Clore J.N. (2001). Nonalcoholic steatohepatitis: Association of insulin resistance and mitochondrial abnormalities. Gastroenterology.

[10] O’Shea R.S., Dasarathy S., McCullough A.J. (2010). Alcoholic liver disease. Hepatology.

[11] Thurman R.G. (1998). Alcoholic liver injury involves activation of Kupffer cells by endotoxin. Am. J. Physiol..

[12] Purohit V., Gao B., Song B.J. (2009). Molecular Mechanisms of Alcoholic Fatty Liver. Alcohol. Clin. Exp. Res..

[13] Szabo G., Mandrekar P. (2009). A recent perspective on alcohol, immunity, and host defense. Alcohol. Clin. Exp. Res..

[14] Gao B., Seki E., Brenner D.A., Friedman S., Cohen J.I., Nagy L., Szabo G., Zakhari S. (2011). Innate immunity in alcoholic liver disease. Am. J. Physiol. Gastrointest. Liver Physiol..

[15] Gao B., Bataller R. (2011). Alcoholic liver disease: Pathogenesis and new therapeutic targets. Gastroenterology.

[16] Holt M.P., Ju C. (2006). Mechanisms of drug-induced liver injury. AAPS J..

[17] Lee W.M. (2003). Drug-induced hepatotoxicity. N. Engl. J. Med..

[18] Hunt C.M., Westerkam W.R., Stave G.M. (1992). Effect of age and gender on the activity of human hepatic CYP3A. Biochem. Pharmacol..

[19] Weinshilboum R. (2003). Inheritance and Drug Response. N. Engl. J. Med..

[20] Guengerich F.P. (2001). Common and uncommon cytochrome P450 reactions related to metabolism and chemical toxicity. Chem. Res. Toxicol..

[21] Zhai Y., Busuttil R.W., Kupiec-Weglinski J.W. (2011). Liver ischemia and reperfusion injury: New insights into mechanisms of innate-adaptive immune-mediated tissue inflammation. Am. J. Transplant..

[22] Gujral J.S., Bucci T.J., Farhood A., Jaeschke H. (2001). Mechanism of cell death during warm hepatic ischemia-reperfusion in rats: Apoptosis or necrosis?. Hepatology.

[23] Rüdiger H.A., Clavien P.A. (2002). Tumor necrosis factor α, but not Fas, mediates hepatocellular apoptosis in the murine ischemic liver. Gastroenterology.

[24] Jaeschke H., Woolbright B.L. (2012). Current strategies to minimize hepatic ischemia-reperfusion injury by targeting reactive oxygen species. Transplant. Rev..

[25] Jaeschke H., Farhood A., Smith C.W. (1990). Neutrophils controbute to ischaemia/reperfusion injury in rat liver in vivo. FASEB J..

[26] Vardanian A.J., Busuttil R.W., Kupiec-Weglinski J.W. (2008). Molecular mediators of liver ischemia and reperfusion injury: A brief review. Mol. Med..

[27] Klune J.R., Tsung A. (2010). Molecular Biology of Liver Ischemia/Reperfusion Injury: Established Mechanisms and Recent Advancements. Surg. Clin. N. Am..

[28] Heneghan M.A., Yeoman A.D., Verma S., Smith A.D., Longhi M.S. (2013). Autoimmune hepatitis. Lancet.

[29] Krawitt E.L. (2006). Autoimmune hepatitis. N. Engl. J. Med..

[30] Manns M.P., Lohse A.W., Vergani D. (2015). Autoimmune hepatitis-Update 2015. J. Hepatol..

[31] Longhi M.S., Liberal R., Holder B., Robson S.C., Ma Y., Mieli-Vergani G., Vergani D. (2012). Inhibition of Interleukin-17 Promotes Differentiation of CD25^–^ Cells Into Stable T Regulatory Cells in Patients With Autoimmune Hepatitis. Gastroenterology.

[32] Liberal R., Grant C.R., Mieli-Vergani G., Vergani D. (2013). Autoimmune hepatitis: A comprehensive review. J. Autoimmun..

[33] Chen G.Y., Nuñez G. (2010). Sterile inflammation: Sensing and reacting to damage. Nat. Rev. Immunol..

[34] Lamkanfi M., Dixit V.M. (2014). Mechanisms and functions of inflammasomes. Cell.

[35] Miao E.A., Rajan J.V., Aderem A. (2011). Caspase-1-induced pyroptotic cell death. Immunol. Rev..

[36] Martinon F., Pétrilli V., Mayor A., Tardivel A., Tschopp J. (2006). Gout-associated uric acid crystals activate the NALP3 inflammasome. Nature.

[37] Muruve D.A., Pétrilli V., Zaiss A.K., White L.R., Clark S.A., Ross P.J., Parks R.J., Tschopp J. (2008). The inflammasome recognizes cytosolic microbial and host DNA and triggers an innate immune response. Nature.

[38] Davis B.K., Wen H., Ting J.P. (2011). The Inflammasome NLRs in Immunity, Inflammation, and Associated Diseases. Annu. Rev. Immunol..

[39] Srinivasula S.M., Poyet J.L., Razmara M., Datta P., Zhang Z., Alnemri E.S. (2002). The PYRIN-CARD protein ASC is an activating adaptor for caspase-1. J. Biol. Chem..

[40] Stehlik C., Lee S.H., Dorfleutner A., Stassinopoulos A., Sagara J., Reed J.C. (2003). Apoptosis-Associated Speck-Like Protein Containing a Caspase Recruitment Domain Is a Regulator of Procaspase-1 Activation. J. Immunol..

[41] Broz P., Dixit V.M. (2016). Inflammasomes: Mechanism of assembly, regulation and signalling. Nat. Rev. Immunol..

[42] Kay E., Scotland R.S., Whiteford J.R. (2014). Toll-like receptors: Role in inflammation and therapeutic potential. BioFactors.

[43] Burnstock G. (2016). P2X ion channel receptors and inflammation. Purinergic Signal..

[44] Ting J.P., Lovering R.C., Alnemri E.S., Bertin J., Boss J.M., Davis B.K., Flavell R.A., Girardin S.E., Godzik A., Harton J.A. (2008). The NLR Gene Family: A Standard Nomenclature. Immunity.

[45] Sha Y., Markovic-Plese S. (2011). A role of IL-1R1 signaling in the differentiation of Th17 cells and the development of autoimmune diseases. Self/Nonself.

[46] Tsutsui H., Cai X., Hayashi S. (2015). Interleukin-1 Family Cytokines in Liver Diseases. Mediat. Inflamm..

[47] Petrasek J., Bala S., Csak T., Lippai D., Kodys K., Menashy V., Barrieau M., Min S.Y., Kurt-Jones E.A., Szabo G. (2012). IL-1 receptor antagonist ameliorates inflammasome-dependent alcoholic steatohepatitis in mice. J. Clin. Investig..

[48] Mridha A.R., Wree A., Robertson A.A.B., Yeh M.M., Johnson C.D., Van Rooyen D.M., Haczeyni F., Teoh N.C., Savard C., Ioannou G.N. (2017). NLRP3 inflammasome blockade reduces liver inflammation and fibrosis in experimental NASH in mice. J. Hepatol..

[49] Szabo G., Csak T. (2012). Inflammasomes in liver diseases. J. Hepatol..

[50] Burnstock G. (2002). Purinergic signaling and vascular cell proliferation and death. Arterioscler. Thromb. Vasc. Biol..

[51] Robson S.C., Sévigny J., Zimmermann H. (2006). The E-NTPDase family of ectonucleotidases: Structure function relationships and pathophysiological significance. Purinergic Signal..

[52] Bours M.J.L., Swennen E.L.R., Di Virgilio F., Cronstein B.N., Dagnelie P.C. (2006). Adenosine 5′-triphosphate and adenosine as endogenous signaling molecules in immunity and inflammation. Pharmacol. Ther..

[53] Katsnelson M.A., Rucker L.G., Russo H.M., Dubyak G.R. (2015). K^+^ efflux agonists induce NLRP3 inflammasome activation independently of Ca2^+^ signaling. J. Immunol..

[54] Kahlenberg J.M., Dubyak G.R. (2004). Mechanisms of caspase-1 activation by P2X7 receptor-mediated K^+^ release. Am. J. Physiol. Cell Physiol..

[55] Cruz C.M., Rinna A., Forman H.J., Ventura A.L.M., Persechini P.M., Ojcius D.M. (2007). ATP activates a reactive oxygen species-dependent oxidative stress response and secretion of proinflammatory cytokines in macrophages. J. Biol. Chem..

[56] Guo H., Callaway J.B., Ting J.P.Y. (2015). Inflammasomes: Mechanism of action, role in disease, and therapeutics. Nat. Med..

[57] Strowig T., Henao-Mejia J., Elinav E., Flavell R. (2012). Inflammasomes in health and disease. Nature.

[58] Chen G.Y., Nez G. (2011). Inflammasomes in intestinal inflammation and cancer. Gastroenterology.

[59] Wree A., Eguchi A., McGeough M.D., Pena C.A., Johnson C.D., Canbay A., Hoffman H.M., Feldstein A.E. (2014). NLRP3 inflammasome activation results in hepatocyte pyroptosis, liver inflammation, and fibrosis in mice. Hepatology.

[60] Boaru S.G., Borkham-Kamphorst E., Tihaa L., Haas U., Weiskirchen R. (2012). Expression analysis of inflammasomes in experimental models of inflammatory and fibrotic liver disease. J. Inflamm..

[61] Watanabe A., Sohail M.A., Gomes D.A., Hashmi A., Nagata J., Sutterwala F.S., Mahmood S., Jhandier M.N., Shi Y., Flavell R.A. (2009). Inflammasome-mediated regulation of hepatic stellate cells. Am. J. Physiol. Gastrointest. Liver Physiol..

[62] He K., Zhu X., Liu Y., Miao C., Wang T., Li P., Zhao L., Chen Y., Gong J., Cai C. (2017). Inhibition of NLRP3 inflammasome by thioredoxin-interacting protein in mouse Kupffer cells as a regulatory mechanism for non-alcoholic fatty liver disease development. Oncotarget.

[63] Csak T., Ganz M., Pespisa J., Kodys K., Dolganiuc A., Szabo G. (2011). Fatty acid and endotoxin activate inflammasomes in mouse hepatocytes that release danger signals to stimulate immune cells. Hepatology.

[64] Wree A., McGeough M.D., Peña C.A., Schlattjan M., Li H., Inzaugarat M.E., Messer K., Canbay A., Hoffman H.M., Feldstein A.E. (2014). NLRP3 inflammasome activation is required for fibrosis development in NAFLD. J. Mol. Med..

[65] Henao-Mejia J., Elinav E., Jin C., Hao L., Mehal W.Z., Strowig T., Thaiss C.A., Kau A.L., Eisenbarth S.C., Jurczak M.J. (2012). Inflammasome-mediated dysbiosis regulates progression of NAFLD and obesity. Nature.

[66] Csak T., Pillai A., Ganz M., Lippai D., Petrasek J., Park J.K., Kodys K., Dolganiuc A., Kurt-Jones E.A., Szabo G. (2014). Both bone marrow-derived and non-bone marrow-derived cells contribute to AIM2 and NLRP3 inflammasome activation in a MyD88-dependent manner in dietary steatohepatitis. Liver Int..

[67] Cai C., Zhu X., Li P., Li J., Gong J., Shen W., He K. (2017). NLRP3 Deletion Inhibits the Non-alcoholic Steatohepatitis Development and Inflammation in Kupffer Cells Induced by Palmitic Acid. Inflammation.

[68] Dixon L.J., Berk M., Thapaliya S., Papouchado B.G., Feldstein A.E. (2012). Caspase-1-mediated regulation of fibrogenesis in diet-induced steatohepatitis. Lab. Investig..

[69] Dixon L.J., Flask C.A., Papouchado B.G., Feldstein A.E., Nagy L.E. (2013). Caspase-1 as a Central Regulator of High Fat Diet-Induced Non-Alcoholic Steatohepatitis. PLoS ONE.

[70] Das S., Seth R.K., Kumar A., Kadiiska M.B., Michelotti G., Diehl A.M., Chatterjee S. (2013). Purinergic receptor X7 is a key modulator of metabolic oxidative stress-mediated autophagy and inflammation in experimental nonalcoholic steatohepatitis. Am. J. Physiol. Gastrointest. Liver Physiol..

[71] Ioannou G.N., Haigh W.G., Thorning D., Savard C. (2013). Hepatic cholesterol crystals and crown-like structures distinguish NASH from simple steatosis. J. Lipid Res..

[72] Coll R.C., Robertson A.A., Chae J.J., Higgins S.C., Muñoz-Planillo R., Inserra M.C., Vetter I., Dungan L.S., Monks B.G., Stutz A. (2015). A small-molecule inhibitor of the NLRP3 inflammasome for the treatment of inflammatory diseases. Nat. Med..

[73] Tilg H., Wilmer A., Vogel W., Herold M., Nölchen B., Judmaier G., Huber C. (1992). Serum levels of cytokines in chronic liver diseases. Gastroenterology.

[74] Dinarello C.A. (2009). Interleukin-1β and the Autoinflammatory Diseases. N. Engl. J. Med..

[75] Cui K., Yan G., Xu C., Chen Y., Wang J., Zhou R., Bai L., Lian Z., Wei H., Sun R. (2015). Invariant NKT cells promote alcohol-induced steatohepatitis through interleukin-1β in mice. J. Hepatol..

[76] Petrasek J., Iracheta-Vellve A., Saha B., Satishchandran A., Kodys K., Fitzgerald K.A., Kurt-Jones E.A., Szabo G. (2015). Metabolic danger signals, uric acid and ATP, mediate inflammatory cross-talk between hepatocytes and immune cells in alcoholic liver disease. J. Leukoc. Biol..

[77] Iracheta-Vellve A., Petrasek J., Satishchandran A., Gyongyosi B., Saha B., Kodys K., Fitzgerald K.A., Kurt-Jones E.A., Szabo G. (2015). Inhibition of sterile danger signals, uric acid and ATP, prevents inflammasome activation and protects from alcoholic steatohepatitis in mice. J. Hepatol..

[78] Heo M.J., Kim T.H., You J.S., Blaya D., Sancho-Bru P., Kim S.G. (2018). Alcohol dysregulates miR-148a in hepatocytes through FoxO1, facilitating pyroptosis via TXNIP overexpression. Gut.

[79] Hoque R., Sohail M.A., Salhanick S., Malik A.F., Ghani A., Robson S.C., Mehal W.Z. (2012). P2X7 receptor-mediated purinergic signaling promotes liver injury in acetaminophen hepatotoxicity in mice. Am. J. Physiol. Gastrointest. Liver Physiol..

[80] Imaeda A.B., Watanabe A., Sohail M.A., Mahmood S., Mohamadnejad M., Sutterwala F.S., Flavell R.A., Mehal W.Z. (2009). Acetaminophen-induced hepatotoxicity in mice is dependent on Tlr9 and the Nalp3 inflammasome. J. Clin. Investig..

[81] Kataoka H., Kono H., Patel Z., Rock K.L. (2014). Evaluation of the contribution of multiple DAMPs and DAMP receptors in cell death-induced sterile inflammatory responses. PLoS ONE.

[82] Williams C.D., Farhood A., Jaeschke H. (2010). Role of caspase-1 and interleukin-1β in acetaminophen-induced hepatic inflammation and liver injury. Toxicol. Appl. Pharmacol..

[83] Kim H.Y., Kim S.J., Lee S.M. (2015). Activation of NLRP3 and AIM2 inflammasomes in Kupffer cells in hepatic ischemia/reperfusion. FEBS J..

[84] Huang H., Chen H.W., Evankovich J., Yan W., Rosborough B.R., Nace G.W., Ding Q., Loughran P., Beer-Stolz D., Billiar T.R. (2013). Histones Activate the NLRP3 Inflammasome in Kupffer Cells during Sterile Inflammatory Liver Injury. J. Immunol..

[85] Kamo N., Ke B., Ghaffari A.A., Shen X.D., Busuttil R.W., Cheng G., Kupiec-Weglinski J.W. (2013). ASC/caspase-1/IL-1β signaling triggers inflammatory responses by promoting HMGB1 induction in liver ischemia/reperfusion injury. Hepatology.

[86] Zhu P., Duan L., Chen J., Xiong A., Xu Q., Zhang H., Zheng F., Tan Z., Gong F., Fang M. (2011). Gene Silencing of NALP3 Protects against Liver Ischemia–Reperfusion Injury in Mice. Hum. Gene Ther..

[87] Inoue Y., Shirasuna K., Kimura H., Usui F., Kawashima A., Karasawa T., Tago K., Dezaki K., Nishimura S., Sagara J. (2014). NLRP3 Regulates Neutrophil Functions and Contributes to Hepatic Ischemia-Reperfusion Injury Independently of Inflammasomes. J. Immunol..

[88] Kato A., Gabay C., Okaya T., Lentsch A.B. (2002). Specific role of interleukin-1 in hepatic neutrophil recruitment after ischemia/reperfusion. Am. J. Pathol..

[89] Zhou R., Tardivel A., Thorens B., Choi I., Tschopp J. (2010). Thioredoxin-interacting protein links oxidative stress to inflammasome activation. Nat. Immunol..

[90] Yue S., Zhu J., Zhang M., Li C., Zhou X., Zhou M., Ke M., Busuttil R.W., Ying Q.L., Kupiec-Weglinski J.W. (2016). The myeloid heat shock transcription factor 1/β-catenin axis regulates NLR family, pyrin domain-containing 3 inflammasome activation in mouse liver ischemia/reperfusion injury. Hepatology.

[91] Zhuang Y., Li Y., Li X., Xie Q., Wu M. (2016). Atg7 knockdown augments concanavalin A-induced acute hepatitis through an ROS-mediated p38/MAPK pathway. PLoS ONE.

[92] Chen K., Li J., Li S., Feng J., Wu L., Liu T., Zhang R., Xu S., Cheng K., Zhou Y. (2016). 15d-PGJ2 alleviates ConA-induced acute liver injury in mice by up-regulating HO-1 and reducing hepatic cell autophagy. Biomed. Pharmacother..

[93] Luan J., Zhang X., Wang S., Li Y., Fan J., Chen W., Zai W., Wang S., Wang Y., Chen M. (2018). NOD-like receptor protein 3 inflammasome-dependent IL-1β accelerated ConA-induced hepatitis. Front. Immunol..

[94] Gong T., Wang X., Yang Y., Yan Y., Yu C., Zhou R., Jiang W. (2017). Plant Lectins Activate the NLRP3 Inflammasome To Promote Inflammatory Disorders. J. Immunol..

[95] Chen L., Lu F.B., Chen D.Z., Wu J.L., Hu E.D., Xu L.M., Zheng M.H., Li H., Huang Y., Jin X.Y. (2018). BMSCs-derived miR-223-containing exosomes contribute to liver protection in experimental autoimmune hepatitis. Mol. Immunol..

[96] Ioannou G.N., Van Rooyen D.M., Savard C., Haigh W.G., Yeh M.M., Teoh N.C., Farrell G.C. (2015). Cholesterol-lowering drugs cause dissolution of cholesterol crystals and disperse Kupffer cell crown-like structures during resolution of NASH. J. Lipid Res..

[97] Li X., Zhang Y., Jin Q., Xia K.L., Jiang M., Cui B.W., Wu Y.L., Song S.Z., Lian L.H., Nan J.X. (2018). Liver kinase B1/AMP-activated protein kinase-mediated regulation by gentiopicroside ameliorates P2X7 receptor-dependent alcoholic hepatosteatosis. Br. J. Pharmacol..

[98] Cai C., Huang H., Whelan S., Liu L., Kautza B., Luciano J., Wang G., Chen G., Stratimirovic S., Tsung A. (2014). Benzyl alcohol attenuates acetaminophen-induced acute liver injury in a Toll-like receptor-4-dependent pattern in mice. Hepatology.

[99] Chiazza F., Couturier-Maillard A., Benetti E., Mastrocola R., Nigro D., Cutrin J.C., Serpe L., Aragno M., Fantozzi R., Ryffel B. (2015). Targeting the NLRP3 inflammasome to reduce diet-induced metabolic abnormalities in mice. Mol. Med..

[100] Puri P., Baillie R.A., Wiest M.M., Mirshahi F., Choudhury J., Cheung O., Sargeant C., Contos M.J., Sanyal A.J. (2007). A lipidomic analysis of nonalcoholic fatty liver disease. Hepatology.

[101] Van Rooyen D.M., Larter C.Z., Haigh W.G., Yeh M.M., Ioannou G., Kuver R., Lee S.P., Teoh N.C., Farrell G.C. (2011). Hepatic free cholesterol accumulates in obese, diabetic mice and causes nonalcoholic steatohepatitis. Gastroenterology.

[102] Savard C., Tartaglione E.V., Kuver R., Haigh W.G., Farrell G.C., Subramanian S., Chait A., Yeh M.M., Quinn L.S., Ioanno G.N. (2013). Synergistic interaction of dietary cholesterol and dietary fat in inducing experimental steatohepatitis. Hepatology.

[103] Duewell P., Kono H., Rayner K.J., Sirois C.M., Vladimer G., Bauernfeind F.G., Abela G.S., Franchi L., Nuñez G., Schnurr M. (2010). NLRP3 inflammasomes are required for atherogenesis and activated by cholesterol crystals. Nature.

[104] Rajamäki K., Lappalainen J., Oörni K., Välimäki E., Matikainen S., Kovanen P.T., Eklund K.K. (2010). Cholesterol crystals activate the NLRP3 inflammasome in human macrophages: A novel link between cholesterol metabolism and inflammation. PLoS ONE.

[105] Cannito S., Morello E., Bocca C., Foglia B., Benetti E., Novo E., Chiazza F., Rogazzo M., Fantozzi R., Povero D. (2017). Microvesicles released from fat-laden cells promote activation of hepatocellular NLRP3 inflammasome: A pro-inflammatory link between lipotoxicity and non-alcoholic steatohepatitis. PLoS ONE.

[106] Tavares De Almeida I., Cortez-Pinto H., Fidalgo G., Rodrigues D., Camilo M.E. (2002). Plasma total and free fatty acids composition in human non-alcoholic steatohepatitis. Clin. Nutr..

[107] Miura K., Yang L., van Rooijen N., Brenner D.A., Ohnishi H., Seki E. (2013). Toll-like receptor 2 and palmitic acid cooperatively contribute to the development of nonalcoholic steatohepatitis through inflammasome activation in mice. Hepatology.

[108] Ricchi M., Odoardi M.R., Carulli L., Anzivino C., Ballestri S., Pinetti A., Fantoni L.I., Marra F., Bertolotti M.M., Banni S. (2009). Differential effect of oleic and palmitic acid on lipid accumulation and apoptosis in cultured hepatocytes. J. Gastroenterol. Hepatol..

[109] DeSantis D.A., Ko C.W., Liu Y., Liu X., Hise A.G., Nunez G., Croniger C.M. (2013). Alcohol-induced liver injury is modulated by Nlrp3 and Nlrc4 inflammasomes in mice. Mediat. Inflamm..

[110] Liu Z.X., Han D., Gunawan B., Kaplowitz N. (2006). Neutrophil depletion protects against murine acetaminophen hepatotoxicity. Hepatology.

[111] Lawson J.A., Farhood A., Hopper R.D., Bajt M.L., Jaeschke H. (2000). The hepatic inflammatory response after acetaminophen overdose: Role of neutrophils. Toxicol. Sci..

[112] Holt M.P., Cheng L., Ju C. (2008). Identification and characterization of infiltrating macrophages in acetaminophen-induced liver injury. J. Leukoc. Biol..

[113] Huebener P., Pradere J.P., Hernandez C., Gwak G.Y., Caviglia J.M., Mu X., Loike J.D., Jenkins R.E., Antoine D.J., Schwabe R.F. (2015). The HMGB1/RAGE axis triggers neutrophil-mediated injury amplification following necrosis. J. Clin. Investig..

[114] Marques P.E., Amaral S.S., Pires D.A., Nogueira L.L., Soriani F.M., Lima B.H., Lopes G.A., Russo R.C., Avila T.V., Melgaço J.G. (2012). Chemokines and mitochondrial products activate neutrophils to amplify organ injury during mouse acute liver failure. Hepatology.

[115] Marques P.E., Oliveira A.G., Pereira R.V., David B.A., Gomides L.F., Saraiva A.M., Pires D.A., Novaes J.T., Patricio D.O., Cisalpino D. (2015). Hepatic DNA deposition drives drug-induced liver injury and inflammation in mice. Hepatology.

[116] Mossanen J.C., Krenkel O., Ergen C., Govaere O., Liepelt A., Puengel T., Heymann F., Kalthoff S., Lefebvre E., Eulberg D. (2016). Chemokine (C-C motif) receptor 2–positive monocytes aggravate the early phase of acetaminophen-induced acute liver injury. Hepatology.

[117] Enari M., Sakahira H., Yokoyama H., Okawa K., Iwamatsu A., Nagata S. (1998). A caspase-activated DNase that degrades DNA during apoptosis, and its inhibitor ICAD. Nature.

[118] Huck S., Deveaud E., Namane A., Zouali M. (1999). Abnormal DNA methylation and deoxycytosine-deoxyguanine content in nucleosomes from lymphocytes undergoing apoptosis. FASEB J..

[119] Cover C., Liu J., Farhood A., Malle E., Waalkes M.P., Bajt M.L., Jaeschke H. (2006). Pathophysiological role of the acute inflammatory response during acetaminophen hepatotoxicity. Toxicol. Appl. Pharmacol..

[120] Williams C.D., Bajt M.L., Farhood A., Jaeschke H. (2010). Acetaminophen-induced hepatic neutrophil accumulation and inflammatory liver injury in CD18-deficient mice. Liver Int..

[121] Bauer I., Vollmar B., Jaeschke H., Rensing H., Kraemer T., Larsen R., Bauer M. (2000). Transcriptional activation of heme oxygenase-1 and its functional significance in acetaminophen-induced hepatitis and hepatocellular injury in the rat. J. Hepatol..

[122] Zhang C., Feng J., Du J., Zhuo Z., Yang S., Zhang W., Wang W., Zhang S., Iwakura Y., Meng G. (2017). Macrophage-derived IL-1α promotes sterile inflammation in a mouse model of acetaminophen hepatotoxicity. Cell. Mol. Immunol..

[123] Woolbright B.L., Jaeschke H. (2016). Role of the Inflammasome in Acetaminophen-induced Liver Injury and Acute Liver Failure. J. Hepatol..

[124] Wanner G.A., Ertel W., Müller P., Höfer Y., Leiderer R., Menger M.D., Messmer K. (1996). Liver ischemia and reperfusion induces a systemic inflammatory response through Kupffer cell activation. Shock.

[125] Åkerfelt M., Morimoto R.I., Sistonen L. (2010). Heat shock factors: Integrators of cell stress, development and lifespan. Nat. Rev. Mol. Cell Biol..

[126] Tiegs G., Hentschel J., Wendel A. (1992). A T cell-dependent experimental liver injury in mice inducible by concanavalin A. J. Clin. Investig..

[127] Erhardt A., Tiegs G. (2010). Tolerance induction in response to liver inflammation. Dig. Dis..

[128] Schümann J., Wolf D., Pahl A., Brune K., Papadopoulos T., van Rooijen N., Tiegs G. (2000). Importance of Kupffer cells for T-cell-dependent liver injury in mice. Am. J. Pathol..

[129] Kawamura H., Aswad F., Minagawa M., Govindarajan S., Dennert G. (2006). P2X7 receptors regulate NKT cells in autoimmune hepatitis. J. Immunol..

[130] Bauernfeind F., Rieger A., Schildberg F.A., Knolle P.A., Schmid-Burgk J.L., Hornung V. (2012). NLRP3 Inflammasome Activity Is Negatively Controlled by miR-223. J. Immunol..

[131] Schmitz J., Owyang A., Oldham E., Song Y., Murphy E., McClanahan T.K., Zurawski G., Moshrefi M., Qin J., Li X. (2005). IL-33, an interleukin-1-like cytokine that signals via the IL-1 receptor-related protein ST2 and induces T helper type 2-associated cytokines. Immunity.

[132] Carriere V., Roussel L., Ortega N., Lacorre D.A., Americh L., Aguilar L., Bouche G., Girard J.P. (2007). IL-33, the IL-1-like cytokine ligand for ST2 receptor, is a chromatin-associated nuclear factor in vivo. Proc. Natl. Acad. Sci. USA.

[133] Cayrol C., Girard J.P. (2018). Interleukin-33 (IL-33): A nuclear cytokine from the IL-1 family. Immunol. Rev..

[134] Bessa J., Meyer C.A., de Vera Mudry M.C., Schlicht S., Smith S.H., Iglesias A., Cote-Sierra J. (2015). Altered subcellular localization of IL-33 leads to non-resolving lethal inflammation. J. Autoimmun..

[135] Lefrançais E., Roga S., Gautier V., Gonzalez-de-Peredo A., Monsarrat B., Girard J.P., Cayrol C. (2012). IL-33 is processed into mature bioactive forms by neutrophil elastase and cathepsin G. Proc. Natl. Acad. Sci. USA.

[136] Chackerian A.A., Oldham E.R., Murphy E.E., Schmitz J., Pflanz S., Kastelein R.A. (2007). IL-1 Receptor Accessory Protein and ST2 Comprise the IL-33 Receptor Complex. J. Immunol..

[137] Villarreal D.O., Weiner D.B. (2014). Interleukin 33: A switch-hitting cytokine. Curr. Opin. Immunol..

[138] Bandara G., Beaven M.A., Olivera A., Gilfillan A.M., Metcalfe D.D. (2015). Activated mast cells synthesize and release soluble ST2-a decoy receptor for IL-33. Eur. J. Immunol..

[139] Moussion C., Ortega N., Girard J.P. (2008). The IL-1-like cytokine IL-33 is constitutively expressed in the nucleus of endothelial cells and epithelial cells in vivo: A novel “Alarmin”?. PLoS ONE.

[140] Pichery M., Mirey E., Mercier P., Lefrancais E., Dujardin A., Ortega N., Girard J.P. (2012). Endogenous IL-33 Is Highly Expressed in Mouse Epithelial Barrier Tissues, Lymphoid Organs, Brain, Embryos, and Inflamed Tissues: In Situ Analysis Using a Novel Il-33-LacZ Gene Trap Reporter Strain. J. Immunol..

[141] Martin N.T., Martin M.U. (2016). Interleukin 33 is a guardian of barriers and a local alarmin. Nat. Immunol..

[142] Liew F.Y., Girard J.P., Turnquist H.R. (2016). Interleukin-33 in health and disease. Nat. Rev. Immunol..

[143] Cayrol C., Girard J.P. (2014). IL-33: An alarmin cytokine with crucial roles in innate immunity, inflammation and allergy. Curr. Opin. Immunol..

[144] Griesenauer B., Paczesny S. (2017). The ST2/IL-33 axis in immune cells during inflammatory diseases. Front. Immunol..

[145] Schiering C., Krausgruber T., Chomka A., Fröhlich A., Adelmann K., Wohlfert E.A., Pott J., Griseri T., Bollrath J., Hegazy A.N. (2014). The alarmin IL-33 promotes regulatory T-cell function in the intestine. Nature.

[146] Siede J., Fröhlich A., Datsi A., Hegazy A.N., Varga D.V., Holecska V., Saito H., Nakae S., Löhning M. (2016). IL-33 receptor-expressing regulatory t cells are highly activated, Th2 biased and suppress CD4 T Cell proliferation through IL-10 and TGFβ Release. PLoS ONE.

[147] Delacher M., Imbusch C.D., Weichenhan D., Breiling A., Hotz-Wagenblatt A., Träger U., Hofer A.C., Kägebein D., Wang Q., Frauhammer F. (2017). Genome-wide DNA-methylation landscape defines specialization of regulatory T cells in tissues. Nat. Immunol..

[148] Salimi M., Barlow J.L., Saunders S.P., Xue L., Gutowska-Owsiak D., Wang X., Huang L.C., Johnson D., Scanlon S.T., McKenzie A.N. (2013). A role for IL-25 and IL-33-driven type-2 innate lymphoid cells in atopic dermatitis. J. Exp. Med..

[149] Monticelli L.A., Osborne L.C., Noti M., Tran S.V., Zaiss D.M.W., Artis D. (2015). IL-33 promotes an innate immune pathway of intestinal tissue protection dependent on amphiregulin–EGFR interactions. Proc. Natl. Acad. Sci. USA.

[150] Monticelli L.A., Sonnenberg G.F., Abt M.C., Alenghat T., Ziegler C.G., Doering T.A., Angelosanto J.M., Laidlaw B.J., Yang C.Y., Sathaliyawala T. (2011). Innate lymphoid cells promote lung-tissue homeostasis after infection with influenza virus. Nat. Immunol..

[151] Kurowska-Stolarska M., Stolarski B., Kewin P., Murphy G., Corrigan C.J., Ying S., Pitman N., Mirchandani A., Rana B., van Rooijen N. (2009). IL-33 Amplifies the Polarization of Alternatively Activated Macrophages That Contribute to Airway Inflammation. J. Immunol..

[152] Matta B.M., Lott J.M., Mathews L.R., Liu Q., Rosborough B.R., Blazar B.R., Turnquist H.R. (2014). IL-33 Is an Unconventional Alarmin That Stimulates IL-2 Secretion by Dendritic Cells To Selectively Expand IL-33R/ST2^+^ Regulatory T Cells. J. Immunol..

[153] Préfontaine D., Lajoie-Kadoch S., Foley S., Audusseau S., Olivenstein R., Halayko A.J., Lemière C., Martin J.G., Hamid Q. (2009). Increased Expression of IL-33 in Severe Asthma: Evidence of Expression by Airway Smooth Muscle Cells. J. Immunol..

[154] Chang Y.J., Kim H.Y., Albacker L.A., Baumgarth N., McKenzie A.N., Smith D.E., Dekruyff R.H., Umetsu D.T. (2011). Innate lymphoid cells mediate influenza-induced airway hyper-reactivity independently of adaptive immunity. Nat. Immunol..

[155] Stolarski B., Kurowska-Stolarska M., Kewin P., Xu D., Liew F.Y. (2010). IL-33 Exacerbates Eosinophil-Mediated Airway Inflammation. J. Immunol..

[156] Tang S., Huang H., Hu F., Zhou W., Guo J., Jiang H., Mu R., Li Z. (2013). Increased IL-33 in synovial fluid and paired serum is associated with disease activity and autoantibodies in rheumatoid arthritis. Clin. Dev. Immunol..

[157] Palmer G., Talabot-Ayer D., Lamacchia C., Toy D., Seemayer C.A., Viatte S., Finckh A., Smith D.E., Gabay C. (2009). Inhibition of interleukin-33 signaling attenuates the severity of experimental arthritis. Arthritis Rheumatol..

[158] Pastorelli L., Garg R.R., Hoang S.B., Spina L., Mattioli B., Scarpa M., Fiocchi C., Vecchi M., Pizarro T.T. (2010). Epithelial-derived IL-33 and its receptor ST2 are dysregulated in ulcerative colitis and in experimental Th1/Th2 driven enteritis. Proc. Natl. Acad. Sci. USA.

[159] Oboki K., Ohno T., Kajiwara N., Arae K., Morita H., Ishii A., Nambu A., Abe T., Kiyonari H., Matsumoto K. (2010). IL-33 is a crucial amplifier of innate rather than acquired immunity. Proc. Natl. Acad. Sci. USA.

[160] Sedhom M.A., Pichery M., Murdoch J.R., Foligné B., Ortega N., Normand S., Mertz K., Sanmugalingam D., Brault L., Grandjean T. (2013). Neutralisation of the interleukin-33/ST2 pathway ameliorates experimental colitis through enhancement of mucosal healing In mice. Gut.

[161] Duan L., Chen J., Zhang H., Yang H., Zhu P., Xiong A., Xia Q., Zheng F., Tan Z., Gong F. (2012). Interleukin-33 ameliorates experimental colitis through promoting Th2/Foxp3^+^ regulatory T-cell responses in mice. Mol. Med..

[162] Turnquist H.R., Zhao Z., Rosborough B.R., Liu Q., Castellaneta A., Isse K., Wang Z., Lang M., Stolz D.B., Zheng X.X. (2011). IL-33 Expands Suppressive CD11b^+^ Gr-1int and Regulatory T Cells, including ST2L^+^ Foxp3^+^ Cells, and Mediates Regulatory T Cell-Dependent Promotion of Cardiac Allograft Survival. J. Immunol..

[163] Reichenbach D.K., Schwarze V., Matta B.M., Tkachev V., Lieberknecht E., Liu Q., Koehn B.H., Pfeifer D., Taylor P.A., Prinz G. (2015). The IL-33/ST2 axis augments effector T-cell responses during acute GVHD. Blood.

[164] Matta B.M., Reichenbach D.K., Zhang X., Mathews L., Koehn B.H., Dwyer G.K., Lott J.M., Uhl F.M., Pfeifer D., Feser C.J. (2016). Peri-alloHCT IL-33 administration expands recipient T-regulatory cells that protect mice against acute GVHD. Blood.

[165] Roth G.A., Zimmermann M., Lubsczyk B.A., Pilz J., Faybik P., Hetz H., Hacker S., Mangold A., Bacher A., Krenn C.G. (2010). Up-regulation of interleukin 33 and soluble ST2 serum levels in liver failure. J. Surg. Res..

[166] McHedlidze T., Waldner M., Zopf S., Walker J., Rankin A.L., Schuchmann M., Voehringer D., McKenzie A.N., Neurath M.F., Pflanz S. (2013). Interleukin-33-dependent innate lymphoid cells mediate hepatic fibrosis. Immunity.

[167] Wang J., Cai Y., Ji H., Feng J., Ayana D.A., Niu J., Jiang Y. (2012). Serum IL-33 levels are associated with liver damage in patients with chronic hepatitis B. J. Interferon Cytokine Res..

[168] Wang J., Zhao P., Guo H., Sun X., Jiang Z., Xu L., Feng J., Niu J., Jiang Y. (2012). Serum IL-33 levels are associated with liver damage in patients with chronic hepatitis C. Mediat. Inflamm..

[169] Marvie P., Lisbonne M., L’helgoualc’h A., Rauch M., Turlin B., Preisser L., Bourd-Boittin K., Théret N., Gascan H., Piquet-Pellorce C. (2010). Interleukin-33 overexpression is associated with liver fibrosis in mice and humans. J. Cell. Mol. Med..

[170] Gao Y., Liu Y., Yang M., Guo X., Zhang M., Li H., Li J., Zhao J. (2016). IL-33 treatment attenuated diet-induced hepatic steatosis but aggravated hepatic fibrosis. Oncotarget.

[171] Pejnovic N., Jeftic I., Jovicic N., Arsenijevic N., Lukic M.L. (2016). Galectin-3 and IL-33/ST2 axis roles and interplay in dietinduced steatohepatitis. World J. Gastroenterol..

[172] Vasseur P., Dion S., Filliol A., Genet V., Lucas-Clerc C., Jean-Philippe G., Silvain C., Lecron J.C., Piquet-Pellorce C., Samson M. (2017). Endogenous IL-33 has no effect on the progression of fibrosis during experimental steatohepatitis. Oncotarget.

[173] Jeftic I., Jovicic N., Pantic J., Arsenijevic N., Lukic M.L., Pejnovic N. (2015). Galectin-3 Ablation Enhances Liver Steatosis, but Attenuates Inflammation and IL-33-Dependent Fibrosis in Obesogenic Mouse Model of Nonalcoholic Steatohepatitis. Mol. Med..

[174] Wang M., Shen G., Xu L., Liu X., Brown J.M., Feng D., Ross R.A., Gao B., Liangpunsakul S., Ju C. (2018). IL-1 receptor like 1 protects against alcoholic liver injury by limiting NF-κB activation in hepatic macrophages. J. Hepatol..

[175] Antunes M.M., Araújo A.M., Diniz A.B., Pereira R.V.S., Alvarenga D.M., David B.A., Rocha R.M., Lopes M.A.F., Marchesi S.C., Nakagaki B.N. (2018). IL-33 signalling in liver immune cells enhances drug-induced liver injury and inflammation. Inflamm. Res..

[176] Yazdani H.O., Chen H.W., Tohme S., Tai S., van der Windt D.J., Loughran P., Rosborough B.R., Sud V., Beer-Stolz D., Turnquist H.R. (2018). IL-33 exacerbates liver sterile inflammation by amplifying neutrophil extracellular trap formation. J. Hepatol..

[177] Sakai N., Van Sweringen H.L., Quillin R.C., Schuster R., Blanchard J., Burns J.M., Tevar A.D., Edwards M.J., Lentsch A.B. (2012). Interleukin-33 is hepatoprotective during liver ischemia/reperfusion in mice. Hepatology.

[178] Yin H., Huang B.J., Yang H., Huang Y.F., Xiong P., Zheng F., Chen X.P., Chen Y.F., Gong F.L. (2006). Pretreatment with soluble ST2 reduces warm hepatic ischemia/reperfusion injury. Biochem. Biophys. Res. Commun..

[179] Liang M., Liwen Z., Yun Z., Yanbo D., Jianping C. (2018). Serum Levels of IL-33 and Correlation with IL-4, IL-17A, and Hypergammaglobulinemia in Patients with Autoimmune Hepatitis. Mediat. Inflamm..

[180] Arshad M.I., Rauch M., L’helgoualc’h A., Julia V., Leite-de-Moraes M.C., Lucas-Clerc C., Piquet-Pellorce C., Samson M. (2011). NKT cells are required to induce high IL-33 expression in hepatocytes during ConA-induced acute hepatitis. Eur. J. Immunol..

[181] Arshad M., Piquet-Pellorce C., L’Helgoualc’h A., Rauch M., Patrat-Delon S., Ezan F., Lucas-Clerc C., Nabti S., Lehuen A., Cubero F.J. (2012). TRAIL but not FasL and TNFα, regulates IL-33 expression in murine hepatocytes during acute hepatitis. Hepatology.

[182] Neumann K., Karimi K., Meiners J., Voetlause R., Steinmann S., Dammermann W., Lüth S., Asghari F., Wegscheid C., Horst A.K. (2017). A Proinflammatory Role of Type 2 Innate Lymphoid Cells in Murine Immune-Mediated Hepatitis. J. Immunol..

[183] Chen J., Duan L., Xiong A., Zhang H., Zheng F., Tan Z., Gong F., Fang M. (2012). Blockade of IL-33 ameliorates Con A-induced hepatic injury by reducing NKT cell activation and IFN-γ production in mice. J. Mol. Med..

[184] Volarevic V., Mitrovic M., Milovanovic M., Zelen I., Nikolic I., Mitrovic S., Pejnovic N., Arsenijevic N., Lukic M.L. (2012). Protective role of IL-33/ST2 axis in Con A-induced hepatitis. J. Hepatol..

[185] Noel G., Arshad M.I., Filliol A., Genet V., Rauch M., Lucas-Clerc C., Lehuen A., Girard J.P., Piquet-Pellorce C., Samson M. (2016). Ablation of interaction between IL-33 and ST2^+^ regulatory T cells increases immune cell-mediated hepatitis and activated NK cell liver infiltration. Am. J. Physiol. Liver Physiol..

[186] Huang H., Tohme S., Al-Khafaji A.B., Tai S., Loughran P., Chen L., Wang S., Kim J., Billiar T., Wang Y. (2015). DAMPs-activated neutrophil extracellular trap exacerbates sterile inflammatory liver injury. Hepatology.

[187] Cayrol C., Girard J.P. (2009). The IL-1-like cytokine IL-33 is inactivated after maturation by caspase-1. Proc. Natl. Acad. Sci. USA.

[188] Lüthi A.U., Cullen S.P., McNeela E.A., Duriez P.J., Afonina I.S., Sheridan C., Brumatti G., Taylor R.C., Kersse K., Vandenabeele P. (2009). Suppression of interleukin-33 bioactivity through proteolysis by apoptotic caspases. Immunity.

[189] Ohno T., Oboki K., Kajiwara N., Morii E., Aozasa K., Flavell R.A., Okumura K., Saito H., Nakae S. (2009). Caspase-1, caspase-8, and calpain are dispensable for IL-33 release by macrophages. J. Immunol..

[190] Carrière V., Arshad M.I., Le Seyec J., Lefevre B., Farooq M., Jan A., Manuel C., Touami-Bernard L., Lucas-Clerc C., Genet V. (2017). Endogenous IL-33 Deficiency Exacerbates Liver Injury and Increases Hepatic Influx of Neutrophils in Acute Murine Viral Hepatitis. Mediat. Inflamm..

[191] Madouri F., Guillou N., Fauconnier L., Marchiol T., Rouxel N., Chenuet P., Ledru A., Apetoh L., Ghiringhelli F., Chamaillard M. (2015). Caspase-1 activation by NLRP3 inflammasome dampens IL-33-dependent house dust mite-induced allergic lung inflammation. J. Mol. Cell. Biol..

[192] Menzel M., Akbarshahi H., Mahmutovic Persson I., Puthia M., Bjermer L., Uller L. (2017). Caspase-1 deficiency reduces eosinophilia and interleukin-33 in an asthma exacerbation model. ERJ Open Res..

[193] Strangward P., Haley M.J., Albornoz M.G., Barrington J., Shaw T., Dookie R., Zeef L., Baker S.M., Winter E., Tzeng T.C. (2018). Targeting the IL33-NLRP3 axis improves therapy for experimental cerebral malaria. Proc. Natl. Acad. Sci. USA.

[194] Zaiss M.M., Maslowski K.M., Mosconi I., Guenat N., Marsland B.J., Harris N.L. (2013). IL-1β suppresses innate IL-25 and IL-33 production and maintains helminth chronicity. PLoS Pathog..

[195] Moulin D., Donzé O., Talabot-Ayer D., Mézin F., Palmer G., Gabay C. (2007). Interleukin (IL)-33 induces the release of pro-inflammatory mediators by mast cells. Cytokine.

